# Human GST P1-1 Redesigned for Enhanced Catalytic Activity with the Anticancer Prodrug Telcyta and Improved Thermostability

**DOI:** 10.3390/cancers16040762

**Published:** 2024-02-12

**Authors:** Aram Ismail, Sridhar Govindarajan, Bengt Mannervik

**Affiliations:** 1 Arrhenius Laboratories, Department of Biochemistry and Biophysics, Stockholm University, SE-10691 Stockholm, Sweden; aram.ismail@dbb.su.se; 2 ATUM, 37950 Central Ct, Newark, CA 94560, USA; sgovindarajan@atum.bio; 3 Department of Chemistry, Scripps Research, La Jolla, CA 92037, USA

**Keywords:** glutathione transferase P1-1, ADEPT, Telcyta, canfosfamide, protein engineering, machine learning, molecular redesign, prodrugs, thermostability

## Abstract

**Simple Summary:**

Antibody-directed enzyme prodrug therapy (ADEPT) is a promising alternative to conventional chemotherapy. The idea is to activate a prodrug into a cytotoxic agent at the tumor site with the aid of an enzyme that has been delivered to the tumor by a therapeutic antibody. Thereby offering reduced off-target toxicity and improved selectivity. Telcyta is a glutathione transferase pi-class (GST P1-1) activated prodrug. The human enzyme has modest activity with Telcyta, although being the most efficient enzyme known. The aim of this work was to molecularly redesign human GST P1-1 for enhanced activity with the prodrug Telcyta to achieve a higher therapeutic index for ADEPT. A single-point mutation improved the activity 2.9-fold over the wildtype value, albeit at the cost of thermal stability. Additional mutations restored stability to GST P1-1 with preserved elevated activity. Our findings represent a first step towards a functional ADEPT application for Telcyta.

**Abstract:**

Protein engineering can be used to tailor enzymes for medical purposes, including antibody-directed enzyme prodrug therapy (ADEPT), which can act as a tumor-targeted alternative to conventional chemotherapy for cancer. In ADEPT, the antibody serves as a vector, delivering a drug-activating enzyme selectively to the tumor site. Glutathione transferases (GSTs) are a family of naturally occurring detoxication enzymes, and the finding that some of them are overexpressed in tumors has been exploited to develop GST-activated prodrugs. The prodrug Telcyta is activated by GST P1-1, which is the GST most commonly elevated in cancer cells, implying that tumors overexpressing GST P1-1 should be particularly vulnerable to Telcyta. Promising antitumor activity has been noted in clinical trials, but the wildtype enzyme has modest activity with Telcyta, and further functional improvement would enhance its usefulness for ADEPT. We utilized protein engineering to construct human GST P1-1 gene variants in the search for enzymes with enhanced activity with Telcyta. The variant Y109H displayed a 2.9-fold higher enzyme activity compared to the wild-type GST P1-1. However, increased catalytic potency was accompanied by decreased thermal stability of the Y109H enzyme, losing 99% of its activity in 8 min at 50 °C. Thermal stability was restored by four additional mutations simultaneously introduced without loss of the enhanced activity with Telcyta. The mutation Q85R was identified as an important contributor to the regained thermostability. These results represent a first step towards a functional ADEPT application for Telcyta.

## 1. Introduction

Cancer is one of the leading causes of death worldwide. Although the survival rate for certain types of cancers can be very high, others have poor prognoses. Traditional cancer chemotherapy has the following limitations: (1) lack of specificity towards malignant cells and (2) drug resistance [1]. Therefore, there is an acute call for more efficacious treatments. Novel approaches such as antibody-directed enzyme prodrug therapy (ADEPT) [2] are designed to overcome both problems. With ADEPT, a drug-activating enzyme is selectively delivered to the tumor site with the aid of a therapeutic antibody, followed by administration of the prodrug. The enzyme used for ADEPT has to fulfill certain key criteria as follows: (1) be able to activate the prodrug, (2) be active and stable under physiological conditions, and (3) not elicit an immune response.

Glutathione transferases (GSTs) are a family of endogenous phase II detoxication enzymes that can be divided into seven classes of cytosolic proteins, namely, Alpha, Mu, Omega, Pi, Sigma, Theta, and Zeta [3]. Primarily, their function is to catalyze the conjugation of electrophilic substrates, including carcinogens, with reduced glutathione (GSH), thereby preventing cellular damage. 

**Figure 1:**
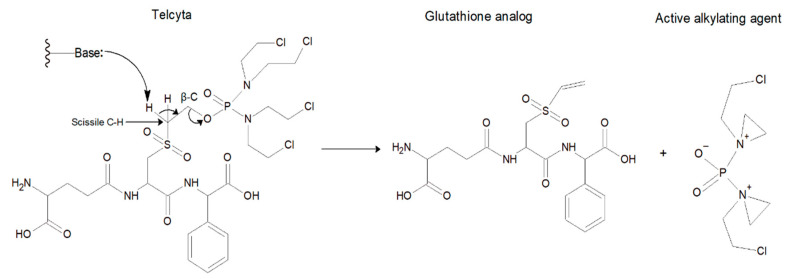
Activation of the prodrug Telcyta. A functional group with a free electron pair can act as a Brønsted base to abstract a proton from a scissile CH_2_ bond through a β-elimination reaction to release the active alkylating agent together with the vinylsulfone glutathione analog.

The pi class in humans is represented by only one member, GST P1-1, which is the most ubiquitously expressed GST and can be found in nearly all tissues apart from the liver. Moreover, GST P1-1 is often overexpressed in various tumor cells, contributing to drug resistance [4,5]. Elevated expression was the basis for developing GST P1-1-activated prodrugs to combat drug-resistant tumors [6,7]. One of these chemotherapeutics is Telcyta (TER286 or canfosfamide), which is a glutathione derivative designed as a prodrug liberating a highly cytotoxic phosphorodiamidate in the presence of GST P1-1 (Figure 1). The phosphorodiamidate released spontaneously undergoes ring-closure into antiproliferative alkylating aziridinium species [7]. The high expression levels of GST P1-1 found in certain tumor cells, coupled with the fact that the enzyme contributes to drug resistance, motivated the design of a prodrug to take advantage of these findings. When the initial work to develop Telcyta started, many derivatives of S-functionalized GSH were evaluated with GSTs known to be expressed in human tumor tissues. In a comparison of alpha, mu, and pi class GSTs, it was determined that Telcyta was preferentially activated by human GST P1-1 [7]. The higher selectivity of GST P1-1 over other GSTs is attributed to the R(-)-phenylglycine moiety of Telcyta (Figure 1), which replaces glycine in the natural GSH tripeptide [7,8]. Telcyta has undergone both phase II and phase III clinical trials [9,10]. In clinical testing for non-small cell lung cancer, breast cancer, as well as ovarian cancer, Telcyta was assessed as a drug with promising antitumor activity, patient tolerance, and relatively low toxicity [11]. 

Although the translational studies recognized Telcyta as a promising anticancer agent, its therapeutic usefulness is limited to tumors with elevated levels of GST P1-1 in comparison with the background of GST P1-1 activity in normal tissues. The therapeutic index can be expected to be raised by ADEPT, and the GST enzyme coupled to antibodies with affinity for tumor-specific epitopes will enable selective delivery to the tumor site irrespective of the intrinsic occurrence of the enzyme. An engineered GST P1-1 with enhanced Telcyta activity can be assumed to increase the rate of Telcyta conversion into active drug molecules, improving the ability to kill the targeted cancer cell and increasing its effect on surrounding tumor mass via bystander activity [12]. Humanized antibodies are established therapeutic agents [13,14,15] and different antibodies can be used to target diverse tumors. GSTs have previously been engineered for increased activity with prodrugs [16], making them highly attractive as drug activating enzymes for ADEPT. Being a human protein, GST P1-1 is not expected to elicit an adverse immune response.

Protein engineering is a powerful tool to design new enzymes with optimized properties, e.g., for medical purposes. However, it can be very difficult to navigate through sequence-space, since the large number of possible sequences for a given protein is 20^n^, where n is the total number of amino acids in the protein, and the vast majority of sequences will not result in a functional protein [17]. The two main strategies in protein engineering are directed evolution and rational design. Directed evolution is based on the selection of enzyme variants with advantageous characteristics from an ensemble of variants. Rational design relies on the structure and/or sequence information of the target enzyme to guide the prediction towards variants with desired properties. In recent years, computer-based methods, such as machine learning, have gained popularity to accelerate the discovery of a functional protein with enhanced properties. One way to navigate protein sequence-function relationships is to use phylogeny and ancestral sequence mapping to build patterns of evolutionary preferred sequence motifs [18], similar to large language models (LLM). Amino acid substitutions found in ancestral nodes of the enzyme class are introduced systematically in designed gene sets so the same substitution appears in multiple variants together with additional substitutions. Consequently, the contribution of individual substitutions (positive, negative, or neutral) and their epistatic effects can be modeled with machine learning to deduce the sequence-activity relationship.

In this investigation, we have enhanced the activity of human GST P1-1 with Telcyta and improved the stability of the enzyme through protein engineering.

## 2. Materials and Methods

### 2.1. Materials

Telcyta,(2S)-2-amino-5-[[(2R)-3-[2-[bis[bis(2-chloroethyl)amino]phosphoryloxy]ethylsulfonyl]-1-[[(R)-carboxy(phenyl)methyl]amino]-1-oxopropan-2-yl]amino]-5-oxopentanoic acid hydrochloride, (CAS: 439943-59-6) was synthesized by Terrapin Technologies Inc. (San Francisco, CA, USA). 1-Chloro-2,4-dinitrobenzene (CDNB) and glutathione (GSH), as well as other chemicals, were purchased from Sigma-Aldrich (Darmstadt, Germany). The plasmid expressing GST P1-1 of the human GSTP1*A gene, encoding the most abundant allelic variant of the enzyme, was available in the laboratory [19]. All the human GST P1-1 variants were based on the same wildtype sequence. Expression clones for mouse and rat GST P1-1, as well as mouse GST P2-2, were synthesized by ATUM (Newark, CA, USA) as previously described for dog GST P1-1 [20].

### 2.2. Protein Expression and Purification

Coding sequences of all GST P1-1 gene variants were optimized by ATUM (Newark, CA) for high-level expression in *Escherichia coli* [21]. All genes were synthesized to contain an N-terminal His_6_-tag to allow for efficient Ni-IMAC purification and inserted into the expression vector pD444-NH (T5-His-ORF, Ecoli-Elec D) by ATUM (Newark, CA, USA). The entire genes of all variants were subjected to Sanger sequencing in order to verify their authenticity. For expression and purification, we followed the protocol of Kolm et al. [22] and the manufacturer’s (Cytiva, Uppsala, Sweden) instructions for Ni-IMAC purification.

All DNA constructs were used to transform *E. coli* BL21 (DE3) cells by a standard heat-shock protocol. The cell suspension was plated on agar plates containing ampicillin (50 µg/mL) and incubated overnight at 37 °C. 50 mL LB medium containing ampicillin (50 µg/mL) was used for overnight growth of a single colony at 37 °C and 200 rpm. 5 mL of cell suspension was used to inoculate 500 mL of 2× YT expression medium (8 g tryptone, 5 g yeast extract, 2.5 g NaCl, and 50 µg/mL ampicillin). The culture was grown at 37 °C and 200 rpm until an OD_600_ of 0.4–0.8 was reached. The log phase culture was induced with isopropyl-1-thio-β-D-galactopyranoside (IPTG) to a final concentration of 0.2 mM and grown for an additional 3 h under the same conditions.

For Ni-IMAC purification, the bacteria pellet was resuspended in cold binding buffer (20 mM sodium phosphate, 20 mM imidazole, 0.5 M NaCl, pH 7.4) supplemented with 0.2 mg/mL chicken egg white lysozyme, 0.2 mM dithiothreitol (DTT), and one Complete Mini protease inhibitor cocktail tablet (Sigma-Aldrich) per 50 mL buffer. The bacteria suspension was incubated on ice for 1 h, cells were disrupted by sonication, and the lysate was centrifuged for 30 min at 27,000× *g*, 4 °C. The crude lysate was applied to the His GraviTrap Ni-IMAC column (Cytiva) after it had been equilibrated with the binding buffer. The column was washed continuously until OD_280_ of the eluate matched the binding buffer. The enzyme was eluted with elution buffer (20 mM sodium phosphate, 0.25 M imidazole, 0.5 M NaCl, pH 7.4) supplemented with 0.2 mM DTT. The purified enzyme was dialyzed against 10 mM Tris-HCl, pH 7.8, containing 1 mM EDTA and 0.2 mM DTT.

Protein concentrations of all purified GST P1-1 samples were estimated by a NanoDrop spectrophotometer, and the molecular mass and homogeneity were estimated by SDS-PAGE.

### 2.3. Activity Measurements with CDNB

Enzyme activity was measured at 30 °C with 1 mM 1-chloro-2,4-dinitrobenzene (CDNB) and 1 mM GSH under standard conditions [23]. The change in absorbance was measured spectrophotometrically at 340 nm in a 1 mL quartz cuvette with 0.1 M sodium phosphate buffer pH 6.5 supplemented with 1 mM EDTA and 0.1% (*w*/*v*) bovine serum albumin. Stock solutions of CDNB were prepared in ethanol (diluted to 5% concentration in cuvette), and GSH was dissolved in the buffer.

### 2.4. Telcyta Calibration Curve

Stock solutions of 20 mM Telcyta were prepared in DMSO, and further dilutions were made in the buffer. A dilution series ranging from 0.4 to 4 mM was constructed. 1 µL of each concentration was applied to a silica-coated thin-layer chromatography plate (Merck, Darmstadt, Germany). The following steps were according to our previously developed procedure [20]. Spot staining was quantified with ImageJ and plotted versus Telcyta concentration. 

### 2.5. Thin-Layer Chromatography to Assay Catalytic Activity of Gst P1-1 Variants with Telcyta

Telcyta is a prodrug composed of a glutathione-like peptide backbone connected to an alkylating agent, and upon activation by GSTs, the cytotoxic agent is released. The glutathione-like moiety of both product and Telcyta is ninhydrin detectable.

The relative catalytic activity of all GST P1-1 variants 37 °C was monitored with the substrate Telcyta by a thin-layer chromatography (TLC) method [20]. The enzyme (0.25 mg/mL) was incubated with 3 mM Telcyta in 0.1 M NaH_2_PO_4_/Na_2_HPO_4_ buffer, pH 7.5. The reaction at 37 °C was monitored over a total of 17.5 min, and samples were withdrawn every 3.5 min starting from zero; 1 µL of reaction mixture was applied to the TLC plate. The mobile phase consisted of Milli-Q water, methanol, acetic acid, and ethyl acetate (ratio: 2:3:3:10). Aliquots of the reaction mixture were analyzed. Following chromatography, the plates were dried for 10 min at 50 °C. For visualization, plates were dipped in ninhydrin solution (0.5% *w*/*v* in methanol) followed by 10 min incubation at 37 °C for ninhydrin staining. ImageJ was used to quantify the size and intensity of the spots representing the formed product and remaining substrate over multiple time points. The non-enzymatic reaction was subtracted from the enzymatic reaction for each time point, and separate graphs were constructed for the starting material and product by plotting time versus spot staining. The catalytic activity was estimated from the slope of the initial linear part of the progress curve.

### 2.6. Thermostability

The thermostability of all GST P1-1 variants with similar catalytic activity as Y109H with Telcyta was investigated at 50 °C by monitoring the decrease in enzyme activity assayed with the substrate CDNB. All enzymes were diluted to equal concentrations (0.2 mg/mL) and incubated in 0.1 M sodium phosphate buffer pH 6.5 at 50 °C. Sampling occurred at different incubation times (0, 2, 4, 8, 16, 32, 64, and 128 min). The activity of each variant was measured spectrophotometrically at 340 nm and 30 °C in the standard CDNB assay, and t_½_, the time at which 50% of the enzyme activity remains, was determined.

### 2.7. Machine Learning Procedure

The machine learning methodology has previously been described in detail [18,24,25,26]. Briefly, the process is divided into the following two steps: Variable selection and Search. Variable selection was performed here by utilizing sequence and structural information to align 100 non-redundant GST P1-1 homologs across phylogeny that share >70% amino acid sequence identity with human GST P1-1. The alignment identifies substitutions that are permissible within the GST family. The identified substitutions are subsequently scored on several different criteria, including distance to seed sequence human P1-1 based on the Dayhoff substitution matrix [27], Principal Component residue clustering [28], Compensatory amino acid covariation [29], and similar. The distribution of each amino acid substitution in each scoring function is mean-centered and, normalized to 1, and summarized across all criteria so that the summarized value can be used to rank the amino acid substitutions. Here, the top 14 amino acid substitutions identified by this ranking were identified and used in the subsequent engineering of GST P1-1.

The search was performed here by using systematic variance so that each of the 14 amino acid substitutions is incorporated in a set of 11 GST P1-1 variants. Each GST P1-1 variant is designed to encode four amino acid substitutions per variant, and every amino acid substitution is present in multiple GST P1-1 variants in accordance with a fractional factorial design algorithm. The factorial design not only captures the functional contribution of each amino acid substitution much more efficiently than one variable at the time but also captures epistatic factors in the underlying protein engineering data.

### 2.8. Homology Modeling

To date, there is no solved crystal structure of GST P1-1 in complex with Telcyta. A crystal structure of human GST P1-1 (PDB code 10GS) with the inhibitory Telcyta analog, TER117, bound to the active site was used to generate a structure with the substitution, Y109H, built in the program MODELLER (version 10.4, University of California San Franscisco, CA, USA) [30] and depicted in Chimera 1.17.1 (version 1.17.1, University of California San Franscisco, CA, USA) [31]. In order to predict a structure with the substitution Q85R, which has been shown to have a positive impact on thermostability, the crystal structure of human wildtype GST P1-1 (PDB code 1PGT) was used.

## 3. Results

### 3.1. Expression and Purification of GST P1-1 Proteins

**Figure 2:**
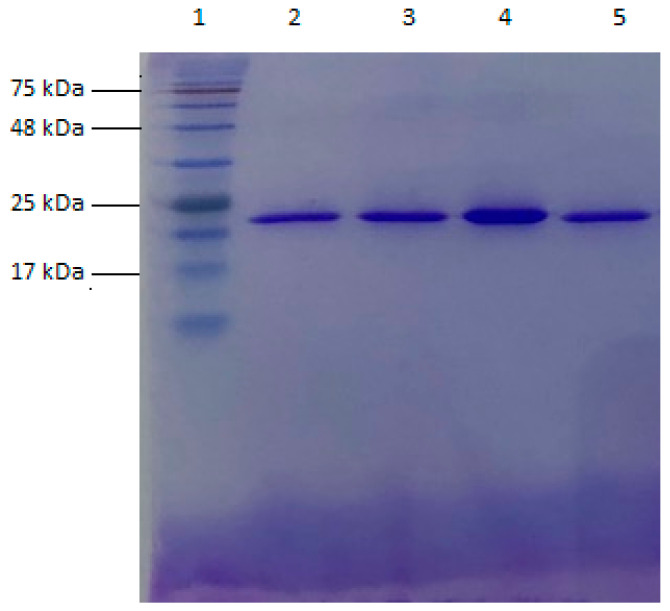
SDS-PAGE analysis of enzyme purity and estimated subunit molecular mass. Lane 1: reference ladder (BLUeye prestained protein ladder, molecular markers ranging from 11 to 245 kDa), lane 2: purified His_6_-tag human GST P1-1 variant Y109H, lane 3: purified His_6_-tag human GST P1-1 variant V6 (Q85R-C102S-S106T-Y109H-V200L), lane 4: purified His_6_-tag human GST P1-1 variant V401 (Y109H-Q85R), and lane 5: purified His_6_-tag wildtype human GST P1-1.Additional electropherograms of purified enzyme variants are shown in Supplemental Materials (Figures S1–S4).

All GST P1-1 variants were expressed in *E. coli* and purified with Ni-IMAC chromatography. The quantities of purified enzyme obtained ranged from 17 to 40 mg per 500 mL growth medium. The homogeneity of each variant was assessed by SDS-PAGE and concluded to be >95%. An analysis of human GST P1-1 variants Y109H, V6 (Q85R-C102S-S106T-Y109H-V200L), V401 (Y109H-Q85R), and wildtype human GST P1-1 is illustrated in Figure 2. The calculated subunit molecular masses of Y109H, V6, Y109H-Q85R, and human P1-1 are 24.152 kDa, 24.192 kDa, 24.180 kDa, and 24.168 kDa, respectively, when the His_6_-tag and initiator methionine are included. 

### 3.2. Telcyta Quantification

**Figure 3:**
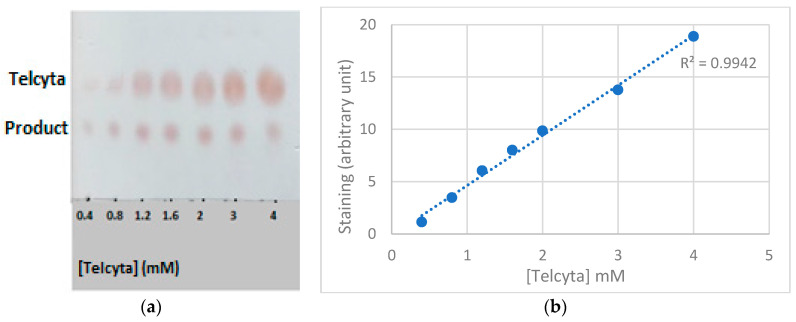
Quantification of Telcyta staining. (**a**) Thin-layer chromatography of Telcyta (upper spots) with concentrations spanning 0.4-4 mM visualized by ninhydrin staining. Lower spots represent the vinylsulfone glutathione analog accompanying phosphorodiamidate release. (**b**) Calibration curve of Telcyta spot staining quantified with ImageJ and plotted against concentration. A linear response was fitted to the data points.

A calibration curve of Telcyta concentrations (0.4–4 mM) developed with ninhydrin staining (Figure 3a) was constructed, and within the selected range, a linear response could be observed (Figure 3b).

### 3.3. Catalytic Activity with Telcyta of Wildtype GST P1-1 Homologs 

CDNB is a standard substrate for determining GST activity, and all purified variants were assayed with CDNB to probe their catalytic competence before testing them with Telcyta.

We assayed naturally existing homologs with Telcyta under conditions described in Section 2.3 and Section 2.5; GST P1-1 and GST P2-2 from mouse as well as GST P1-1 from rat were tested. GST P1-1 from dog had previously been assayed with Telcyta [20]. Out of all tested homologs, the human enzyme was found to be the most active with Telcyta, followed by GST P1-1 from mouse, rat, and dog, respectively. However, mouse GST P2-2 did not show any detectable activity (Table 1).

### 3.4. Site-Directed Point Mutations of Human GST P1-1 

To enhance the activity of human GSTP1-1 with the prodrug Telcyta we initially applied a rational design approach using site-specific mutagenesis. The choice of sites for mutagenesis was mainly based on a previously published model structure with Telcyta docked to the active site of human GST P1-1 [32]. For the first round of mutagenesis we identified two sites, and three single-point mutations were constructed, namely, Y8H, Y8E, and Y109H and their catalytic activities were measured (Table 1). The most prominent activity was found with the variant Y109H, where tyrosine had been substituted with histidine. The activity with Telcyta was estimated to be 2.9-fold higher than that of wild-type human GST P1-1 (Table 1). Moreover, both substitutionsY8H and Y8E had a deleterious effect on activity with Telcyta as well as with CDNB (Table 1).

For the second round of mutagenesis we added three sites, 9, 11, and 36, and a total of 13 double-point mutations were constructed and analyzed (Table 2). The second-generation gene variants showed no improved function (Table 2). However, a number of variants demonstrated a negative effect of mutations added to Y109H on the Telcyta activity, namely, Y109H-F9H and Y109H-V11S/E/H. Although the variants did not have any detectable activity with Telcyta, they were still catalytically competent with CDNB (Table 2).

### 3.5. Designed Variants of Human GST P1-1 for Machine-Learning

To make larger jumps across the fitness landscape, a machine-learning procedure was adopted [18]. The starting point was the Y109H variant, and for this approach homologous sequences of the target protein were examined. A protein BLAST of wildtype human GST P1-1 was performed to find homologous sequences [33]. The top 100 non-redundant sequences with >70% sequence identity were selected for an alignment to identify evolutionarily variable sites and all the naturally occurring amino acid variation within each position. The naturally occurring substitutions were scored based on pre-selected parameters [26]. A total of 17 variants were constructed based on the 14 most frequent substitutions in 11 different positions identified in the homologous sequences. For the first-generation variants, the substitutions were systematically introduced in 11 variants with 4 mutations each. This set of variants was specifically designed to explore the sequence space around the starting sequence, constrained by the 14 substitutions identified by evolutionary analysis of the protein family. 

The GST P1-1 variants were expressed and purified for assays of activity with Telcyta. Three variants indicated improved activity (Table A1, Appendix A), variant V6 was quantified as 3.1 times the activity of the wildtype enzyme (Table 1), but statistically not significantly higher than that of Y109H. For the second generation of variants, we designed the following: one variant (encoding 5 amino acid substitutions) and five gene variants (with 6 or 7 amino acid substitutions each). No variant was found to significantly improve the catalytic activity with Telcyta (Table A2, Appendix A).

**Figure 4:**
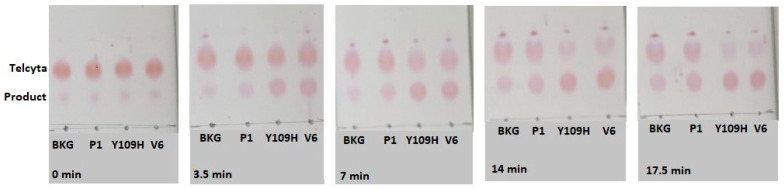
TLC-experiment illustrating the activity of human GST P1-1 (P1) and two variants (Y109H and V6) with the prodrug Telcyta (**upper spots**). Telcyta is enzymatically cleaved into an active alkylating agent and a ninhydrin-detectable vinylsulfone glutathione analog (**lower spots**). Enzyme (0.25 mg/mL) was incubated at pH 7.5 with 3 mM Telcyta. BKG represents the reaction in the absence of enzyme. The reaction at 37 °C was monitored over a total of 17.5 min, and samples were withdrawn every 3.5 min starting from zero; 1 µL of reaction mixture was applied to the plate.

The performance of the most active variants from each engineering approach is specified in Table 1. The Telcyta activation promoted by each variant, exemplified by one experiment, is visualized in Figure 4, wildtype human GST P1-1 serving as a reference.

Approximate Telcyta activity, scored with the human eye, of all variants can be found in Table 2 and Appendix A Table A1 and Table A2.

### 3.6. Thermostability

**Figure 5:**
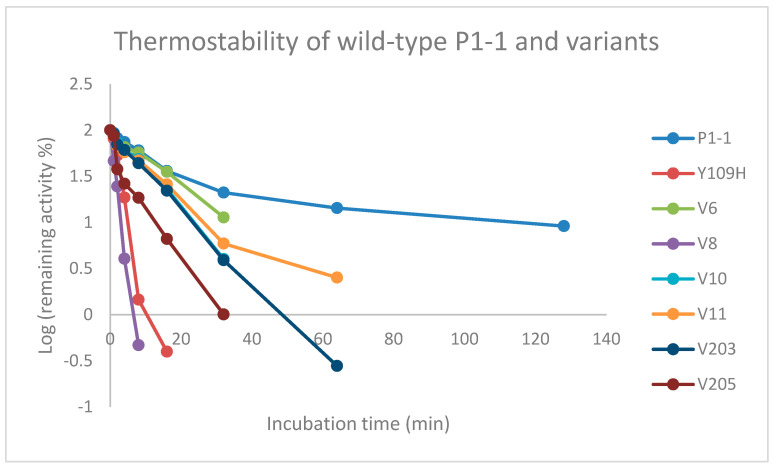
Thermal inactivation of human GST P1-1 variants at 50 °C monitored by remaining catalytic activity with CDNB. Activity in samples taken at different time points was measured in the standard assay system at 30 °C. N.B. The V10 values are overlapped with those of V203.

The overall stability is an important factor directly related to the usefulness of the enzyme. For therapeutic applications, the enzyme needs to be stable under physiological conditions with a half-life long enough to exert a biological effect. Enhancing the catalytic function without impacting the stability is a challenging task, as the introduction of mutations in the enzyme may cause a decrease in stability [34]. Based on these considerations, the thermal stability of variants with similar Telcyta activity as Y109H was investigated by measuring the residual enzyme activity with CDNB at 50 °C (Table 3). Replacing the tyrosine in position 109 with a histidine was found to significantly decrease the thermal stability, thus increasing the rate of inactivation (Table 3). Furthermore, in comparison to wildtype GST P1-1, a shift from second-order decay to first-order decay can be observed (Figure 5). After 8 min, only 1% of the activity of Y109H remained, in contrast to 58% of the wildtype GST P1-1 activity. 

Notably, the machine-learning library generated destabilizing, neutral, and stabilizing mutations with altered half-lives (Table 3) relative to the Y109H variant. The greatest loss of thermal stability was observed for the gene variant V8 (Q40L-E41Q-Q84P-Y109H-V200A). The gene variant that was found to be most thermostable was V6 (Q85R, C102S, S106T, V200L), and the estimated half-life was similar to that of human wildtype GST P1-1 (Table 3). A predictive model was built based on the measured half-lives of all first-generation variants, and the calculated weights of each amino acid substitution were estimated. The substitution Q85R was identified as a contributor to the improved thermostability of variant V6. The Q85R substitution was derived from the variable selection described in the Machine Learning Procedure (Section 2.7). In the amino acid sequence alignment of the top 100 GST P1-1 homologs, residue 85 is represented by glutamine (Q) 36 times, by arginine (R) 60 times, and by lysine (K) 4 times. The substitution also ranks among the top 14 based on all the scoring criteria [26]. To further elucidate the role of the amino acid in position 85, we constructed a gene variant with both substitutions Y109H and Q85R. This gene variant, V401 (Q85R-Y109H), exhibited ~50% of the thermal stability of V6 (Table 3).

### 3.7. Homology Modeling

**Figure 6:**
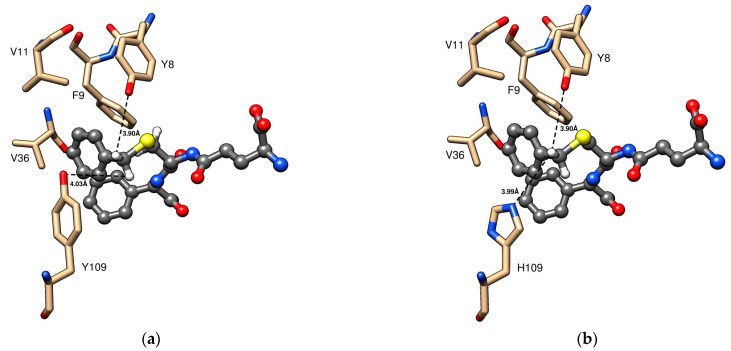
H-site residues in the H-site of GST P1-1 targeted by mutagenesis. (**a**) Close-up of the inhibitor TER117 (γ-L-glutamyl-S-(benzyl)-L-cysteinyl-R(-)-phenylglycine) bound to GST P1-1 based on the crystal structure (PDB code 10GS). The peptide moiety of TER117 is identical to that of Telcyta, but the S-benzyl group differs from the bis-(2-chloroethyl)amino[phosphoryloxy]ethylsulfonyl substituent of Telcyta (Figure 1). The benzylic CH_2_ of TER117 is in the same position as the scissile CH_2_ of Telcyta, which is the chemical group attacked by the enzyme. (**b**) A model of GST P1-1 variant Y109H based on the structure of the wild-type enzyme.

We generated structures in Chimera and MODELLER (University of California, San Franscisco, CA, USA) [30,31] based on published crystal structures of human GST P1-1 in complex with the Telcyta-related inhibitor TER117 (Figure 6; PDB code 10GS) and S-hexyl-glutathione (PDB code 1PGT). Figure 6a displays the closeness of the phenolic hydroxyl groups of both Tyr8 and Tyr109 in wildtype GST P1-1 to the protons of TER117 corresponding to the scissile CH_2_ in Telcyta. The positions of Phe9, Val11, and Val36 also subjected to mutagenesis are similarly indicated. Figure 6b visualizes the new histidine residue in variant Y109H based on modelling of the crystal structure. The imidazole ring of His109 is located at approximately the same distance as the phenol group of Tyr109. The actual reaction trajectory of the proton abstracted from CH_2_ in Telcyta is proposed to involve water molecules in the active site [32]. 

**Figure 7:**
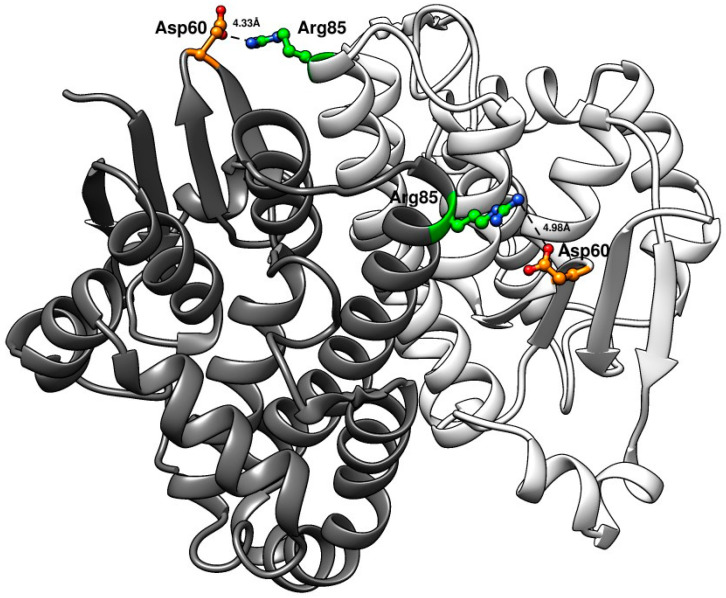
Structure of human GST P1-1 variant V401 (Q85R-Y109H). The two subunits are identical. Arg85 (green) in either subunit interacts with Asp60 (orange) in the neighboring subunit. The model was generated in MODELLER based on the crystal structure of human GST P1-1 (PDB code 1PGT).

Figure 7 shows the modelled structure of the stabilized dimeric human GST P1-1 variant V401 (Q85R-Y109H). The two interacting subunits are identical and illustrate the proposed electrostatic interaction between Arg85 (green) in one subunit formed with Asp60 (orange) in the neighboring subunit. The sidechains can occur as different rotamers, and two possible poses stabilizing the interaction between the two subunits are shown. The distances between the charges of the residues of the opposing subunits are between 4 and 5Å. A similar configuration of Asp60 in relation to the naturally occurring Arg85 in mouse GST P1-1 is shown in its crystal structure (PDB code 8C5D) [35].

## 4. Discussion

### 4.1. Examination of the Enzymatic Activity of Natural GST P1-1 Homologs against Substrate Telcyta

In order to obtain a favorable therapeutic index, it is desirable to obtain a recombinant enzyme more active than the wildtype GST P1-1. For this purpose, we wanted first to investigate if naturally occurring human GST P1-1 homologs would be more active with Telcyta than the human enzyme. The rationale being that discovery of a more efficient homolog could identify residues with a positive influence on catalytic activity, which could aid the redesign of human GST P1-1. 

The human enzyme is a dimeric protein, like other soluble GSTs, with two identical subunits each synthesized with 210 amino acid residues (the initiator Met1 is normally eliminated in the mature enzyme). Each subunit is composed of two domains; a 76-residue long N-terminal domain linked to the 127-residue C-terminal domain [36]. Topologically the catalytic center of the enzyme involves three segments of the primary structure: N-terminal, middle, and C-terminal. The residues binding GSH in a highly specific manner, the G-site, include Tyr8, Arg14, Trp39, Lys45, Gln52, Leu53, Ser66, and Asp99 (the last from the adjacent subunit) [8]. All residues except Asp99 belong to the N-terminal domain. The H-site, responsible for binding different electrophilic substrates, is formed by 8 residues. Asn205 and Gly206 are located in the C-terminal segment, and Ile105 and Tyr109 [37] are found in a helix in the middle segment of domain II in the structure. The remaining H-site residues Tyr8, Phe9, Val11, Arg14 reside in the N-terminal segment. Notably, Tyr8 and Arg14 contribute to both H-site and G-site. Residue 105 is polymorphic, and the most common GSTP1*A allele, Ile105, is used in our investigation. The second most frequently occurring allele, GSTP1*B, encodes Val105, which provides an enzyme that is not more active with several alternative substrates [19]. 

**Figure 8:**
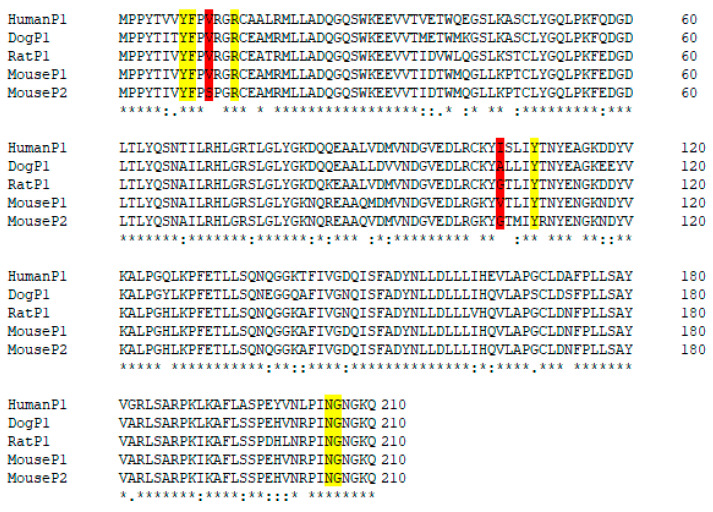
Multiple sequence alignment of human, dog, rat, and mouse class pi GST sequences. H-site residues are highlighted in color. Marked in red are the H-site residues, positions 11 and 105, that are variable among these homologs.

The pi-class homologs from mouse (GST P1-1and P2-2), rat, and dog share high sequence identity with the human enzyme, 85.2%, 83.8%, 85.7%, and 87.2%, respectively. Among these species, the majority of active site residues are conserved, variations are seen only in positions 11 and 105 (Figure 8).

None of the GSTs from the other species showed higher catalytic activity with Telcyta than human GST P1-1, even though all enzymes except mouse GST P2-2 demonstrated some activity (Table 1). Mouse GST P2-2 has Ser in position 11, whereas all other sequences have Val in this position. The second most active enzyme was mouse GST P1-1, which has 97% sequence identity with GST P2-2. Previous research demonstrated that mouse GST P1-1 and GST P2-2 with alternative substrates display major catalytic differences, which are ascribed to a few key residues in positions 11, 12, and 105 [38]. Substituting either Val to Gly in position 105 or Val to Ser in position 11 of mouse GST P1-1 suppressed the catalytic efficiency. By contrast, the simultaneous substitution of Gly11 to Val and Pro12 to Arg in GST P2-2 augmented its catalytic activity by 100-fold. The GST P1-1 enzymes from the other species all contained Val in position 11. The human enzyme has Ile in position 105 (or Val in the allelic variant), mouse GST P1-1 Val, dog GST P1-1 Ala, while rat GST P1-1 and mouse GST P2-2 have Gly. 

Studies on the human enzyme have shown that the enzymes encoded by the two most common allelic variants GSTP1*A (Ile105) and GSTP1*B (Val105), which together account for over 90% of the polymorphism seen in the general population, are equally effective in Telcyta activation [39]. Even though none of the natural homologs tested was found to be more active with Telcyta than the human counterpart, the results demonstrated that Ile or Val in position 105 was superior to Ala or Gly and that Val in position 11 also favored the desired activity. 

In general, mutations outside the active site can alter the substrate specificity and catalytic activity as exemplified in, e.g., subtilisin and β-glycosidase [40,41]. To move forward with our work, we proceeded with the engineering of human GST P1-1, also including residues beyond the active site.

### 4.2. Engineering of Human GST P1-1 and Catalytic Activity with Telcyta

**Figure 9:**
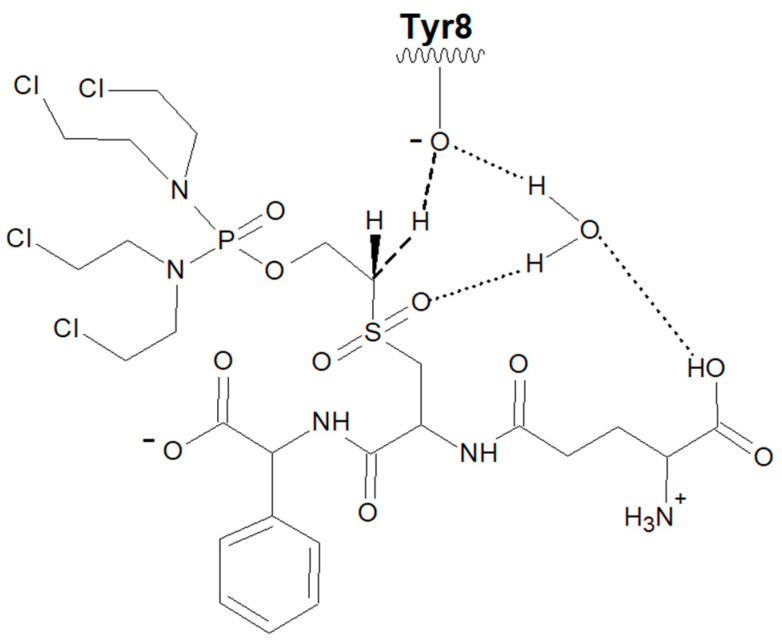
Proposed activation of the prodrug Telcyta by wildtype GST P1-1. A water-assisted proton transfer takes place from the Tyr8 sidechain tyrosyl group to the N-terminal carboxyl group of Telcyta. The deprotonated Tyr8 then abstracts a proton from the scissile carbon of Telcyta [32].

Telcyta activation involves a Brønsted base abstracting a proton from the α-carbon, thereby initiating cleavage of Telcyta into the active alkylating phosphorodiamidate [7] (Figure 1). The base could theoretically be any functional group in the enzyme capable of accepting a proton, and we tested a few alternatives (see below). Crystal structures of wildtype human GST P1-1 reveal the presence of an active-site Tyr8 linked to water molecules [42]. On the basis of computational studies, a water molecule plays a crucial role in the reaction by forming a network of intermolecular interactions between the active-site Tyr8 hydroxyl and the sulfone and COO− groups of Telcyta. The proposed mechanism can be divided into three partial reactions [32]. In the first step, a water molecule transfers a proton between the phenolic Tyr8 hydroxyl and the COO− oxygen atom of Telcyta. In the second step, Tyr8 serves as a base and receives a proton from the scissile C-H bond of Telcyta. In the third step, the bond between the adjacent β-C and the oxygen of the phosphorodiamidate is broken, resulting in the release of the active alkylating agent (Figure 9). The water molecule provides a network of intermolecular connections among the Tyr7 hydroxyl, the Telcyta sulfone, and α-COO− functional groups, which is essential to preserving an effective charge distribution in the different steps of the mechanism. Obviously, the water molecule plays a pivotal role in the reaction. The computational analysis shows that proton transfer is critically dependent on the hydrogen bonding and that no activation of Telcyta takes place in the absence of the water molecule [32].

In the present investigation, enhanced catalytic activity of human GST P1-1 with Telcyta was attempted by creating a more efficient base in the enzyme. The published mechanism [32] involves a crucial role of Tyr8, which is corroborated by our finding that neither variants Y8E nor Y8H showed any activity with Telcyta (Table 1). A variety of point mutations close to the assumed binding site of Telcyta were constructed, and their effect on the catalytic activity was measured (Table 1). The rationale was that the basic form of a residue sidechain of, e.g., histidine, glutamic acid, or aspartic acid could facilitate a proton-abstraction reaction [43], either by assisting Tyr8 or by offering an alternative reaction trajectory. Histidine represents one of the most common active site residues found in enzymes [44], making it a natural choice. Furthermore, the pKa of the imidazolium ion is close to 7, giving it significant contributions of both acidic and basic forms under physiological conditions. The carboxylate of Glu or Asp in a hydrophobic environment could also serve as a base. However, the single-point mutations Y8E and Y8H did not produce any measurable catalytic activity with Telcyta. By contrast, Y109H increased the Telcyta activation approximately 2.9-fold over the wildtype activity. Examination of the structure of TER117 in the active site of GST P1-1 (Figure 6a) indicates the proximity of Tyr109 to the scissile C-H bond of the structurally related substrate Telcyta and suggests that the phenolic hydroxyl group may promote the reaction. His in position 109 could provide an imidazole group supporting catalysis via an alternative functional group (Figure 6b). Even though Tyr109 is an evolutionarily highly conserved active site residue in the hydrophobic binding pocket of GST P1-1 from different biological species, it is replaced by His in grey mouse lemur (*Microcebus murinus*) and horse (*Equus caballus*), suggesting that His may fulfill a functional role in natural homologs. 

The contribution of residue 109 to the catalytic mechanism is almost certainly indirect since the distance to the scissile C-H is too long for direct contact (Figure 6a,b). The peptide moiety is rigidly bound to the G-site of the enzyme and would not undergo conformational changes, as evidenced by the observation that S-hexylglutathione bound to the same site (PDB code 1PGT) overlaps TER117 to the CH_2_ beyond S in the cysteine sidechain. Possibly, both Tyr and His in position 109 could contribute to catalysis by proton transfer via water molecules in the active site. This proposal is supported by the structure of the wildtype GST P1-1 showing the hydroxyl group of Tyr109 hydrogen bonded to a water molecule, which in turn is hydrogen bonded to a second water molecule interacting with Tyr8 and glutathione in the active site [42].

Previous studies have examined GST P1-1 with Tyr109 mutated into Phe [45] and Val [46], demonstrating that residue 109 influences catalytic activity and substrate selectivity. Depending on the substrate used, the catalytic efficiency can be either suppressed or enhanced. Furthermore, Tyr109 is also implicated as a residue of functional importance based on its selective covalent modification with certain GST P1-1 inhibitors [47]. 

Tyr8 in class pi, and the corresponding Tyr residues in alpha, mu, and sigma, is a crucial actor in GSH activation [48], and when Tyr8 in GST P1-1 is mutated to Phe a 300-fold decrease in catalytic efficiency (kcat/Km^GSH^) is observed [49]. Our finding that replacement of Tyr8 by either His or Glu caused a loss in activity shows that these residues cannot adopt the role of Tyr8 in the catalytic mechanism, neither with Telcyta nor with CDNB (Table 1). 

The N-terminal domain of human GST P1-1 is folded like thioredoxin in a motif of secondary structures: β1-α1-β2-α2-β3-β4-α3. The α2-helix (residues 35-46), which is part of the glutathione binding site, has been proven to be flexible [50,51]. This segment of the structure was also targeted for mutagenesis, as fluctuations in orientation may promote catalysis. Within this region, based on the distance to the substrate molecule, Val36 was identified as a suitable site for mutagenesis. Val11 and Phe9 were also identified as candidates due to their proximity to the site of catalysis. 

A second generation of GST P1-1 variants was made in order to find out if the activity of the Y109H mutation could be enhanced by an additional point mutation in positions 9, 11, or 36. Activities with Telcyta were visually scored in comparison to wildtype GST P1-1, and the activity with CDNB was assayed spectrophotometrically in parallel in order to monitor general catalytic competence (Table 2, and Appendix A Table A1 and Table A2). A selection of charged, polar, uncharged, and nonpolar aliphatic residues were paired with the Y109H substitution. All seven double-point mutations involving position 36 were active with Telcyta, even though none showed enhanced activity compared to variant Y109H. However, substituting either Gly or Thr for Val11 decreased the activity close to the wildtype GST P1-1 value (Table 2). The CDNB activity remained similar or at least 50% of the Y109H value. The change of Phe9 to His in Y109H eliminated the enzyme activity with Telcyta, even though low but significant CDNB activity was found. The substitution of the H-site residue Val11 with either His, Ser, or Glu also extinguished the Telcyta activity in parallel with a drastic reduction of CDNB activity (Table 2). Double mutations V11A-Y109H and V11T-Y109H showed very low but measurable Telcyta activation in the same way. Phe9 and Val11 are hydrophobic residues, and both are part of the H-site. Val11, as mentioned before, has been identified as a critical residue responsible for the catalytic function of mouse GST P1-1 [38]. Our finding that substituting Ser for Val in position 11 is in accord with the results of the same substitution in the mouse enzyme. However, it is not clear if the observed decrease in activity in the human V11S-Y109H variant is dependent exclusively on the V11S point mutation due to the presence of His109 or if epistatic interactions operate between residues Ser11 and His109.

### 4.3. Redesign of Gst P1-1 via Machine-Learning Libraries

The two main strategies for enzyme engineering are rational design and directed evolution. Regardless of approach, there is more than one way to introduce mutations in the parent gene [52], and which method to select is a non-trivial issue. However, in cases where resources are scarce, and high-throughput screening is lacking, selecting substitutions that can be predicted beforehand to be favorable or at least neutral is a feasible approach for the exploration of sequence space surrounding the parental protein. A helpful source of information that can guide the exploration of such sequences is the primary structure of naturally occurring protein homologs. These sequences reveal evolutionarily conserved residues within the protein family associated with folding, stability, and functionality. In parallel, non-conserved residues in the primary structures indicate positions that can tolerate changes during evolution and potentially optimize protein functions and create new activities. The combination of the conserved residues in a parental sequence with synthetic variants with substitutions in the variable positions enables the construction of libraries in the multidimensional sequence matrix where proteins can fold into stable and functionally active structures. Such libraries contain information about the desired properties, which can be extracted by analyses of small samples of individual variants [18]. A fractional factorial design, in which a small number of positions in the sequence matrix are varied in different combinations, such that each of the chosen variable positions occurs several times in different sequence contexts allows regression analysis and model building, which provide quantitative information based on the outcome of the selected substitutions. Machine-learning can then aid design and execution of new rounds of gene synthesis and testing of improved variants. This approach makes the exploration of functional space by a small number of sequence-defined variants possible. This approach has previously been successfully applied to engineer enzymes for improved functionality by identifying advantageous residues [18,26,53].

In the present investigation, the machine-learning approach was adopted in an attempt to further enhance its catalytic activity with Telcyta. In parallel the analysis was used in an attempt to increase the stability of the Y109H variant of human GST P1-1. A total of 17 variants were constructed (Table A1 and Table A2, Appendix A). All variants were tested with Telcyta as substrate, but no variant significantly reduced or improved the activity further relative to the Y109H variant. 

### 4.4. Thermostability

It is well established that protein engineering for altered functional properties can be accompanied by loss of stability and other physical properties [34]. Loss of stability may jeopardize the usefulness of an engineered enzyme, e.g., in medical applications such as ADEPT, where the drug-activating enzyme must be stable and active under physiological conditions. Accordingly, we measured the thermostability (Table 3) of catalytically improved GST P1-1 variants. The mutation Y109H was found to be highly destabilizing as compared to wildtype human GST P1-1. It is known that hydrophobic cores are vital for overall protein stability, and introducing substitutions that alter the hydrophobicity are likely to affect the stability. Another factor that can impact stability is sidechain size, i.e., substituting large-to-small residue and vice versa [54]. This is the case in the allelic variants of human GST P1-1 in which the H-site residue Ile105 affords higher stability than Val105 [19]. The observed loss in thermal stability of the Y109H variant reflected in the measured half-life (Table 3), can possibly be attributed to factors such as change in polarity, sidechain size, and hydrogen bonding. Substituting the polar histidine residue for the nonpolar tyrosine residue causes a slight decrease in hydrophobicity. Furthermore, histidine being smaller than tyrosine, thus occupying less volume in the internal cavity, may cause destabilization and, in addition, could also alter the hydrogen binding pattern, including active-site water molecules. The most thermostable enzyme variant, V6 (Q85R-C102S-S106T-Y109H-V200L), displayed a half-life similar to that of wild-type GST P1-1 (Table 3). However, examination of Figure 5 suggests that wild-type GST P1-1 follows a second-order decay, whereas V6 (Q85R-C102S-S106T-Y109H-V200L) follows a first-order process, meaning that wild-type GST P1-1 is more stable over longer periods of time. This is in accordance with previous research, which has shown that the unfolding of wild-type GST P1-1 is a multistep process [55]. 

To understand the increased thermostability of V6, a predictive model was constructed based on the measured half-lives of the GST P1-1 variants. Modeling suggested Q85R to have a beneficial effect. The new variant, V401 (Q85R-Y109H), had a 2-fold increased half-life compared to Y109H and reached 50% of the half-life of V6 (Table 3), thus supporting the role of Arg85 as a contributor to the enzyme stability. Inter-subunit interactions have been shown to enhance thermostability via ion pair bonds [56]. Arg85 is located on the surface of the α4-helix and projects towards Asp60 in the neighboring subunit. The observed increase in thermostability can likely be ascribed to an electrostatic interaction between Arg85 and Asp60 in the adjacent chains (Figure 7). However, it cannot be concluded that the increased half-life of V6 is the result of a single dominant mutation, but it is rather a combined effect of multiple stabilizing amino acid substitutions.

ADEPT is a powerful treatment strategy against cancer with the ability to amplify the therapeutic effect and minimize the side effects of cytotoxic drugs. Although clinical trials are limited (see review) [57], the results are promising, with a big obstacle being immunogenicity of the native enzyme. As mentioned, GSTs are naturally occurring endogenous enzymes and our designed variant, with enhanced Telcyta activity and improved thermostability, only differs in a few residues compared to the human wildtype enzyme and is therefore unlikely to trigger an immune reaction upon administration. The continued work will involve the construction of fusion proteins comprising GST P1-1 and antibody proteins such as a single-chain variable fragment (scFv), e.g., a binder of CD123, which is overexpressed in multiple hematolymphoid neoplasms. We foresee a combinatorial potential such that an array of antibodies or other specific binding proteins directed against different epitopes of various cancers can be linked to GST P1-1 for similar ADEPT.

## 5. Conclusions

The current work demonstrates that human GST P1-1 is the most active homolog with the prodrug Telcyta among the tested wildtype enzymes. Moreover, we used protein engineering to improve the catalytic activity of human GST P1-1 with the prodrug Telcyta as well as the thermostability of the enzyme. A single-point mutation in position 109 improved the catalytic activity 2.9-fold with the substrate, albeit with a significant destabilizing effect. However, an enzyme variant with regained thermostability and upheld activity with Telcyta was obtained by introducing four mutations in addition to that in position 109. Our findings have therapeutic potential as a first step to developing an antibody-directed enzyme prodrug therapy treatment based on Telcyta. 

## Figures and Tables

**Table 1:** Catalytic activity of natural homologs and human GST P1-1 variants with the prodrug Telcyta and the common substrate CDNB relative to wildtype human GST P1-1. The specific activity of human GST P1-1 was previously reported as 106 µmol min^−1^∙mg^−1^ with CDNB [19], whereas the absolute value for Telcyta is unknown. Measurements were made in triplicate with S.D. < 30% for Telcyta and <10% for CDNB. ND = No detectable activity.

	Activity Relative to Wildtype GST P1-1	
Variants	Telcyta (Fold)	CDNB (µmol min^−1^ mg^−1^)
Human P1-1	1	106 ± 4
Mouse P1-1	0.20 ± 0.06	76 ± 2.7
Mouse P2-2	ND	0.10 ± 0.007
Rat P1-1	+	17 ± 1.0
Dog P1-1 ^1^	+	23 ± 1.3
Human P1-1 variant Y8H	ND	0.08 ± 0.004
Human P1-1 variant Y8E	ND	ND
Human P1-1 variant Y109H	2.9 ± 0.6	20.9 ± 0.7
Human P1-1 variant V6 (Q85R-C102S-S106T-Y109H-V200L)	3.1 ± 0.9	28.2 ± 0.3

^1^ Data from [20].

**Table 2:** Enzyme variants from the rationally designed library and their catalytic activities with Telcyta and the common substrate CDNB relative to the parental human GST P1-1. Telcyta activity (scored by + signs) was scored with the human eye. ND = No detectable activity.

	Estimated Activity Relative to Wildtype GST P1-1	
Variants	Telcyta (Score)	CDNB (µmol min^−1^ mg^−1^)
Human P1-1	+++	106 ± 4
Y109H	++++	20.9 ± 0.7
Y8H	ND	0.08 ± 0.004
Y8E	ND	N/D
F9H-Y109H	ND	0.71 ± 0.01
V11H-Y109H	ND	0.01 ± 0.001
V11A-Y109H	+	17.5 ± 0.4
V11S-Y109H	ND	1.82 ± 0.02
V11T-Y109H	+	5.2 ± 0.2
V11E-Y109H	ND	0.027 ± 0.002
V36R-Y109H	+++	20.6 ± 1.2
V36M-Y109H	++++	24.4 ± 1.1
V36G-Y109H	+++	12.4 ± 0.3
V36L-Y109H	++++	22.0 ± 0.3
V36K-Y109H	++++	27.0 ± 2.5
V36I-Y109H	++++	18.5 ± 0.2
V36T-Y109H	++++	15.6 ± 0.2

**Table 3:** Half-lives of wild-type GST P1-1, Y109H and all variant enzymes that displayed similar catalytic activity with Telcyta as Y109H. The variant Y109H-Q85R is a second-generation variant constructed after evaluation of the half-lives of the first-generation variants. Enzyme inactivation was monitored by remaining catalytic activity with CDNB. Activity was measured in the standard assay system at 30 °C.

Substrate: CDNB	
P1-1 Variants	t_½_ (min)
Human P1-1	9.1
Y109H	2.4
V36T-Y109H	2.8
V36L-Y109H	2.3
V1 (T35S-Q40L-A46S-Q85R-Y109H)	2.9
V2 (Q40M-E41Q-A46S-Y109H-V200L)	1.1
V3 (Q40L-S43P-Q85K-Y109H-V200L)	1.7
V4 (T35S-E41Q-Q85K-S106T-Y109H)	2.9
V5 (Q40M-S43P-Q85R-Y109H-S185C)	5.9
V6 (Q85R-C102S-S106T-Y109H-V200L)	10.9
V7 (A46S-S106T-Y109H-S185C-V200A)	4.1
V8 (Q40L-E41Q-Q84P-Y109H-V200A)	0.94
V9 (T35S-S43P-C102S-Y109H-V200A)	1.3
V10 (Q40M-Q84P-Q85K-C102S-Y109H)	7.3
V11 (T35S-Q84P-Y109H-S185C-V200L)	6.7
V201 (T35S-Q40L-E41Q-Q84P-Q85K-S106T-Y109H)	1.4
V202 (T35S-Q40L-E41Q-Q85K-S106T-Y109H-S185C)	2.1
V203 (T35S-E41Q-Q84P-Q85K-S106T-Y109H-S185C)	6.5
V204 (T35S-Q40L-E41Q-Q84P-Q85K-S106T-Y109H-S185C)	1.8
V205 (E41Q-Q84P-Q85K-S106T-Y109H-S185C)	6.8
V206 (Q40L-E41Q-Q84P-Q85P-S106T-Y109H-S185C)	2.3
V401 (Q85R-Y109H)	5.6

## Data Availability

The data reported in this article are available upon reasonable request from the corresponding author.

## Supplementary Materials

The following supporting information can be downloaded at: https://www.mdpi.com/article/10.3390/cancers16040762/s1, Figures S1–S4 showing SDS-PAGE of purified enzyme variants.

## Appendix A

**Table A1:** First generation GST P1-1 variants of the machine-learning library, based on the parent Y109H, and their estimated catalytic activity with Telcyta and the common substrate CDNB compared to wildtype human GST P1-1. Specific CDNB activities determined in triplicate (S.D. < 5%). Relative Telcyta activity (+) scored by eye.

	Estimated Activity Relative to Wildtype GST P1-1	
Variants	Telcyta (Score)	CDNB (µmol min^−1^ mg^−1^)
Human P1-1	+++	106 ± 4
V1 (T35S-Q40L-A46S-Q85R-Y109H)	++++	20.5 ± 1.2
V2 (Q40M-E41Q-A46S-Y109H-V200L)	++++	19.2 ± 0.7
V3 (Q40L-S43P-Q85K-Y109H-V200L)	++++	20.6 ± 0.5
V4 (T35S-E41Q-Q85K-S106T-Y109H)	++++(+)	20.7 ± 0.6
V5 (Q40M-S43P-Q85R-Y109H-S185C)	++++(+)	17.5 ± 0.5
V6 (Q85R-C102S-S106T-Y109H-V200L)	++++(+)	28.2 ± 0.3
V7 (A46S-S106T-Y109H-S185C-V200A)	++++	19.7 ± 0.2
V8 (Q40L-E41Q-Q84P-Y109H-V200A)	++++	21.3 ± 1.3
V9 (T35S-S43P-C102S-Y109H-V200A)	++++	17.9 ± 1.1
V10 (Q40M-Q84P-Q85K-C102S-Y109H)	++++	20.3 ± 0.4
V11 (T35S-Q84P-Y109H-S185C-V200L)	++++	21.9 ± 0.3
V401 (Q85R-Y109H)	++++	20.2 ± 0.7

**Table A2:** GST P1-1 variants from the second-generation machine-learning library, based on the parent Y109H, and their estimated catalytic activities with Telcyta relative to human GST P1-1 and their specific CDNB activities. Specific CDNB activities determined in triplicate (S.D. < 5%). Relative Telcyta activity (+) scored by eye.

	Estimated Activity Relative to Wildtype GST P1-1	
Variants	Telcyta (Score)	CDNB (µmol min^−1^ mg^−1^)
Human P1-1	+++	106 ± 4
V201 (T35S-Q40L-E41Q-Q84P-Q85K-S106T-Y109H)	++++	22.4 ± 1.1
V202 (T35S-Q40L-E41Q-Q85K-S106T-Y109H-S185C)	++++	22.2 ± 1.3
V203 (T35S-E41Q-Q84P-Q85K-S106T-Y109H-S185C)	++++	14.0 ± 0.2
V204 (T35S-Q40L-E41Q-Q84P-Q85K-S106T-Y109H-S185C)	++++	14.1 ± 0.5
V205 (E41Q-Q84P-Q85K-S106T-Y109H-S185C)	++++	13.1 ± 1.0
V206 (Q40L-E41Q-Q84P-Q85P-S106T-Y109H-S185C)	++++	14.6 ± 0.4
