# Supplementary material for: Human GST P1-1 Redesigned for Enhanced Catalytic Activity with the Anticancer Prodrug Telcyta and Improved Thermostability
Source: Cancers (Basel). 2024 Feb 12;16(4):762. doi: 10.3390/cancers16040762 (PMC10887215; doi:10.3390/cancers16040762)
Supplement: Supplementary file 1 [file cancers-16-00762-s001.zip › Figures S1-S4.pdf]

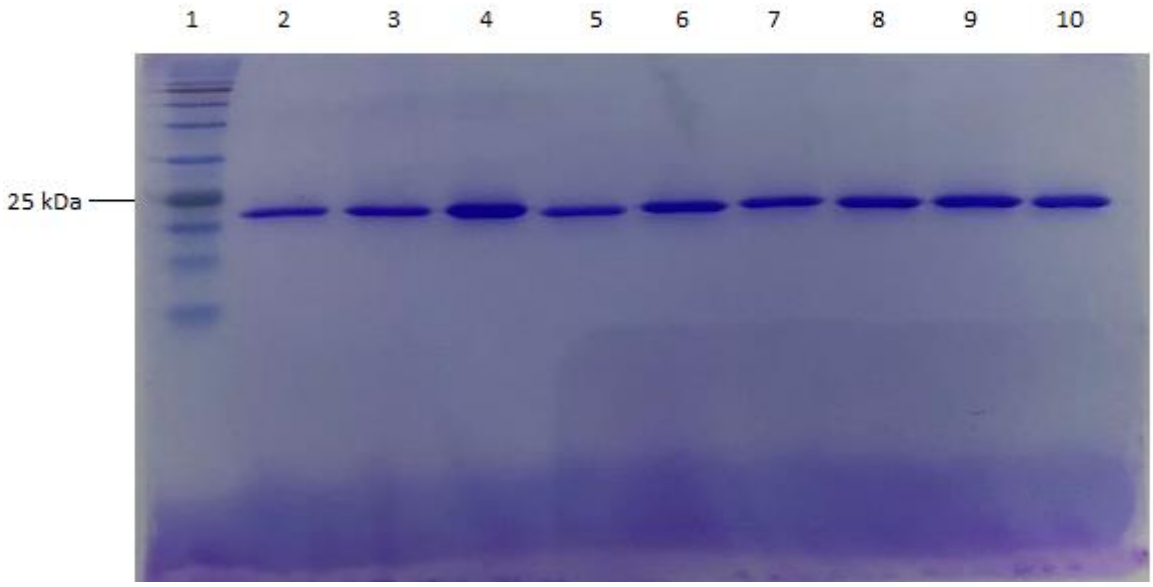

Figure S1. SDS-PAGE analysis of enzyme purity and estimated subunit molecular mass. Lane 1: reference ladder (BLUeye prestained protein ladder, molecular markers ranging from 11 to 245 kDa), lane 2: purified His$_6$-tag human GST P1-1 variant Y109H, lane 3: purified His$_6$-tag human GST P1-1 variant V6 (Q85R-C102S-S106T-Y109H-V200L), lane 4: purified His$_6$-tag human GST P1-1 variant Y109H-Q85R, lane 5: purified His$_6$-tag wildtype human GST P1-1, lane 6: purified His$_6$-tag human GST P1-1 variant V1 (T35S-Q40L-A46S-Q85R-Y109H), lane 7: purified His$_6$-tag human GST P1-1 variant V2 (Q40M-E41Q-A46S-Y109H-V200L), lane 8: purified His$_6$-tag human GST P1-1 variant V3 (Q40L-S43P-Q85K-Y109H-V200L), lane 9: purified His$_6$-tag human GST P1-1 variant V4 (T35S-E41Q-Q85K-S106T-Y109H), and lane 10: purified His$_6$-tag human GST P1-1 variant V5 (Q40M-S43P-Q85R-Y109H-S185C).

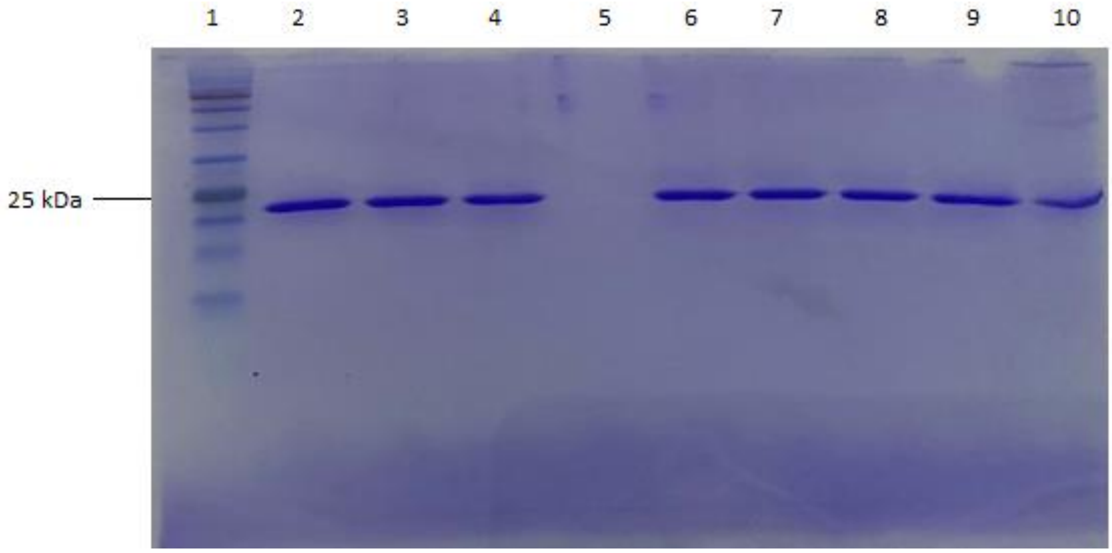

Figure S2. SDS-PAGE analysis of enzyme purity and estimated subunit molecular mass. Lane 1: reference ladder (BLUeye prestained protein ladder, molecular markers ranging from 11 to 245 kDa), lane 2: purified His$_6$-tag human GST P1-1 variant V7 (A46S-S106T-Y109H-S185C-V200A), lane 3: purified His$_6$-tag human GST P1-1 variant V8 (Q40L-E41Q-Q84P-Y109H-V200A), lane 4: purified His$_6$-tag human GST P1-1 variant V9 (T35S-S43P-C102S-Y109H-V200A), lane 5: empty, lane 6: purified His$_6$-tag human GST P1-1 variant V11 (T35S-Q84P-Y109H-S185C-V200L), lane 7: purified His$_6$-tag human GST P1-1 variant V201 (T35S-Q40L-E41Q-Q84P-Q85K-S106T-Y109H), lane 8: purified His$_6$-tag human GST P1-1 variant V202 (T35S-Q40L-E41Q-Q85K-S106T-Y109H-S185C), lane 9: purified His$_6$-tag human GST P1-1 variant V203 (T35S-E41Q-Q84P-Q85K-S106T-Y109H-S185C), and lane 10: purified His$_6$-tag human GST P1-1 variant V205 (E41Q-Q84P-Q85K-S106T-Y109H-S185C).

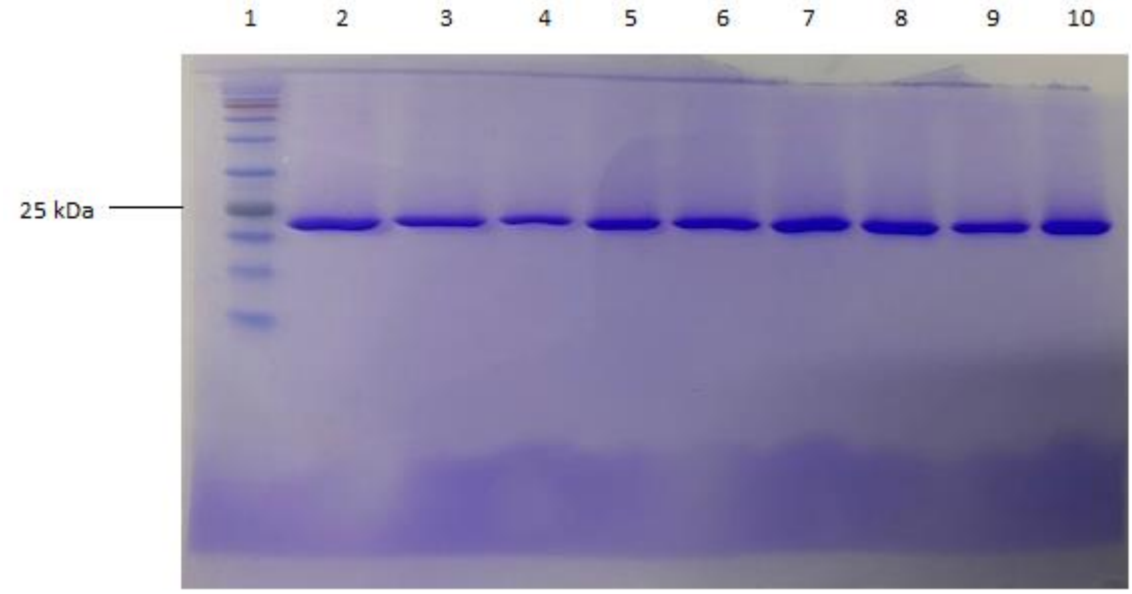

Figure S3. SDS-PAGE analysis of enzyme purity and estimated subunit molecular mass. Lane 1: reference ladder (BLUeye prestained protein ladder, molecular markers ranging from 11 to 245 kDa), lane 2: purified His_6-tag human GST P1-1 variant V204 (T35S-Q40L-E41Q-Q84P-Q85K-S106T-Y109H-S185C), lane 3: purified His_6-tag human GST P1-1 variant V206 (Q40L-E41Q-Q84P-Q85P-S106T-Y109H-S185C), lane 4: purified His_6-tag human GST P1-1 variant Y8E, lane 5: purified His_6-tag human GST P1-1 variant Y8H, lane 6: purified His_6-tag human GST P1-1 variant Y109H-F9H, lane 7: purified His_6-tag human GST P1-1 variant Y109H-V11A, lane 8: purified His_6-tag human GST P1-1 variant Y109H-V11E, lane 9: purified His_6-tag human GST P1-1 variant Y109H-V11H, and lane 10: purified His_6-tag human GST P1-1 variant Y109H-V11T.

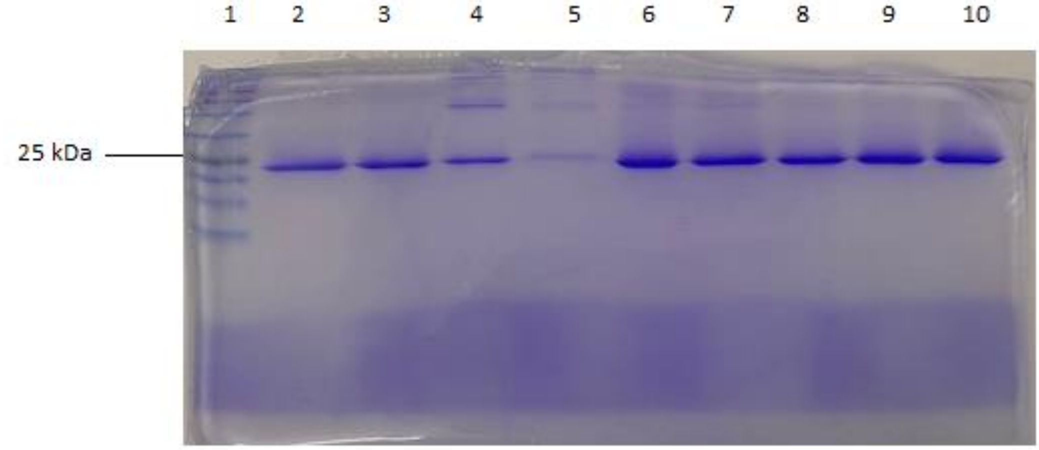

Figure S4. SDS-PAGE analysis of enzyme purity and estimated subunit molecular mass. Lane 1: reference ladder (BLUeye prestained protein ladder, molecular markers ranging from 11 to 245 kDa), lane 2: purified His_6-tag human GST P1-1 variant Y109H-V36R, lane 3: purified His_6-tag human GST P1-1 variant Y109H-V36G, lane 4: purified His_6-tag rat GST P1-1, lane 5: purified His_6-tag mouse GST P2-2, lane 6: purified His_6-tag mouse GST P1-1, lane 7: purified His_6-tag dog GST P1-1, lane 8: purified His_6-tag human GST P1-1 variant Y109H-V36M, lane 9: purified His_6-tag human GST P1-1 variant Y109H-V36T, and lane 10: purified His_6-tag human GST P1-1 variant Y109H-V36K.
