# Supplementary material for: Human GST P1-1 Redesigned for Enhanced Catalytic Activity with the Anticancer Prodrug Telcyta and Improved Thermostability
Source: Cancers (Basel). 2024 Feb 12;16(4):762. doi: 10.3390/cancers16040762 (PMC10887215; doi:10.3390/cancers16040762)
Supplement: Supplementary file 1 [file cancers-16-00762-s001.zip › Sequences.pdf]

Wildtype human GST P1-1

ATGCCCCCCTACACCGTGGTGTACCACCCCGTGAGGGGCAGGTGCGCCGCCCTGAGGATGCTG
CTGGCCGACCAGGGCCAGAGCTGGAAGGAGGAGGTGGTGACCGTGGAGACCTGGCAGGAGGG
CAGCCTGAAGGCCAGCTGCCTGTACGGCCAGCTGCCCAAGTTCCAGGACGGCGACCTGACCCT
GTACCAGAGCAACACCATCCTGAGGCACCTGGGCAGGACCCTGGGCCTGTACGGCAAGGACCA
GCAGGAGGCCGCCCTGGTGGACATGGTGAACGACGGCGTGGAGGACCTGAGGTGCAAGTACAT
CAGCCTGATCTACACCAACTACGAGGCCGGCAAGGACGACTACGTGAAGGCCCTGCCCGGCCA
GCTGAAGCCCTTCGAGACCCTGCTGAGCCAGAACCAGGGCGGCAAGACCTTCATCGTGGGCGA
CCAGATCAGCTTCGCCGACTACAACCTGCTGGACCTGCTGCTGATCCACGAGGTGCTGGCCCCC
GGCTGCCTGGACGCCTTCCCCCTGCTGAGCGCCTACGTGGGCAGGCTGAGCGCCAGGCCCAAG
CTGAAGGCCTTCCTGGCCAGCCCCGAGTACGTGAACCTGCCCATCAACGGCAACGGCAAGCAG

Y109H

ATGCCACCATATACCGTTGTCTATTTCCCTGTCCGTGGCCGCTGTGCGGCGCTGCGTATGCTGTTGGC
CGACCAGGGTCAGAGCTGGAAAGAAGAAGTTGTTACCGTTGAAACGTGGCAAGAGGGCTCTCTGAAA
GCGAGCTGCCTGTACGGCCAACTGCCGAAGTTTCAGGACGGTGACCTGACGTTGTACCAGAGCAAC
ACCATCCTGCGTCACTTGGGTCGCACGTTGGGTCTGTACGGTAAGGACCAGCAAGAGGCCGCACTG
GTGGACATGGTTAACGATGGTGTCGAGGATCTGCGCTGCAAATACATCAGCCTGATCCATACTAACTAT
GAGGCCGGTAAAGATGATTACGTGAAAGCGCTGCCGGGTCAACTGAAGCCGTTTGAAACCCTGTTGA
GCCAGAACCAGGGTGGCAAGACCTTCATTGTGGGCGATCAAATTTCGTTCGCAGACTATAATCTGCTG
GACCTGCTGCTGATCCACGAAGTGCTGGCTCCGGGCTGTCTGGATGCATTTCCGCTGCTGAGCGCTT
ACGTGGGCCGTCTGAGCGCGCGTCCGAAGCTGAAGGCGTTCTTGGCATCCCCGGAGTATGTCAATC
TGCCGATTAATGGTAATGGTAAACAATAATGA

Y8E

AGGAGGTAAAACATATGCACCACCATCACCATCATCCACCATATACCGTTGTCGAGTTCCCTGTCCGT
GGCCGCTGTGCGGCGCTGCGTATGCTGTTGGCCGACCAGGGTCAGAGCTGGAAAGAAGAAGTTGTT
ACCGTTGAAACGTGGCAAGAGGGCTCTCTGAAAGCGAGCTGCCTGTACGGCCAACTGCCGAAGTTT
CAGGACGGTGACCTGACGTTGTACCAGAGCAACACCATCCTGCGTCACTTGGGTCGCACGTTGGGT
CTGTACGGTAAGGACCAGCAAGAGGCCGCACTGGTGGACATGGTTAACGATGGTGTCGAGGATCTG
CGCTGCAAATACATCAGCCTGATCTATACTAACTATGAGGCCGGTAAAGATGATTACGTGAAAGCGCTG
CCGGGTCAACTGAAGCCGTTTGAAACCCTGTTGAGCCAGAACCAGGGTGGCAAGACCTTCATTGTG
GGCGATCAAATTTCGTTCGCAGACTATAATCTGCTGGACCTGCTGCTGATCCACGAAGTGCTGGCTCC
GGGCTGTCTGGATGCATTTCCGCTGCTGAGCGCTTACGTGGGCCGTCTGAGCGCGCGTCCGAAGCT
GAAGGCGTTCTTGGCATCCCCGGAGTATGTCAATCTGCCGATTAATGGTAATGGTAAACAATAATGACT
CGAG

Y8H

ATGCCACCATATACCGTTGTCCATTTCCCTGTCCGTGGCCGCTGTGCGGCGCTGCGTATGCTGTTGG
CCGACCAGGGTCAGAGCTGGAAAGAAGAAGTTGTTACCGTTGAAACGTGGCAAGAGGGCTCTCTGA
AAGCGAGCTGCCTGTACGGCCAACTGCCGAAGTTTCAGGACGGTGACCTGACGTTGTACCAGAGCA
ACACCATCCTGCGTCACTTGGGTCGCACGTTGGGTCTGTACGGTAAGGACCAGCAAGAGGCCGCAC
TGGTGGACATGGTTAACGATGGTGTCGAGGATCTGCGCTGCAAATACATCAGCCTGATCTATACTAACT
ATGAGGCCGGTAAAGATGATTACGTGAAAGCGCTGCCGGGTCAACTGAAGCCGTTTGAAACCCTGTT

GAGCCAGAACCAGGGTGGCAAGACCTTCATTGTGGGCGATCAAATTTCGTTCGCAGACTATAATCTGC
TGGACCTGCTGCTGATCCACGAAGTGCTGGCTCCGGGCTGTCTGGATGCATTTCCGCTGCTGAGCG
CTTACGTGGGCCGTCTGAGCGCGCGTCCGAAGCTGAAGGCGTTCTTGGCATCCCCGGAGTATGTCA
ATCTGCCGATTAATGGTAATGGTAAACAATAATGA

F9H-Y109H

ATGCCACCATATACCGTTGTCTATCATCCTGTCCGTGGCCGCTGTGCGGCGCTGCGTATGCTGTTGGC
CGACCAGGGTCAGAGCTGGAAAGAAGAAGTTGTTACCGTTGAAACGTGGCAAGAGGGCTCTCTGAAA
GCGAGCTGCCTGTACGGCCAACTGCCGAAGTTTCAGGACGGTGACCTGACGTTGTACCAGAGCAAC
ACCATCCTGCGTCACTTGGGTCGCACGTTGGGTCTGTACGGTAAGGACCAGCAAGAGGCCGCACTG
GTGGACATGGTTAACGATGGTGTCGAGGATCTGCGCTGCAAATACATCAGCCTGATCCATACTAACTAT
GAGGCCGGTAAAGATGATTACGTGAAAGCGCTGCCGGGTCAACTGAAGCCGTTTGAAACCCTGTTGA
GCCAGAACCAGGGTGGCAAGACCTTCATTGTGGGCGATCAAATTTCGTTCGCAGACTATAATCTGCTG
GACCTGCTGCTGATCCACGAAGTGCTGGCTCCGGGCTGTCTGGATGCATTTCCGCTGCTGAGCGCTT
ACGTGGGCCGTCTGAGCGCGCGTCCGAAGCTGAAGGCGTTCTTGGCATCCCCGGAGTATGTCAATC
TGCCGATTAATGGTAATGGTAAACAATAATGA

V11H-Y109H

ATGCCACCATATACCGTTGTCTATTTCCCTCATCGTGGCCGCTGTGCGGCGCTGCGTATGCTGTTGGC
CGACCAGGGTCAGAGCTGGAAAGAAGAAGTTGTTACCGTTGAAACGTGGCAAGAGGGCTCTCTGAAA
GCGAGCTGCCTGTACGGCCAACTGCCGAAGTTTCAGGACGGTGACCTGACGTTGTACCAGAGCAAC
ACCATCCTGCGTCACTTGGGTCGCACGTTGGGTCTGTACGGTAAGGACCAGCAAGAGGCCGCACTG
GTGGACATGGTTAACGATGGTGTCGAGGATCTGCGCTGCAAATACATCAGCCTGATCCATACTAACTAT
GAGGCCGGTAAAGATGATTACGTGAAAGCGCTGCCGGGTCAACTGAAGCCGTTTGAAACCCTGTTGA
GCCAGAACCAGGGTGGCAAGACCTTCATTGTGGGCGATCAAATTTCGTTCGCAGACTATAATCTGCTG
GACCTGCTGCTGATCCACGAAGTGCTGGCTCCGGGCTGTCTGGATGCATTTCCGCTGCTGAGCGCTT
ACGTGGGCCGTCTGAGCGCGCGTCCGAAGCTGAAGGCGTTCTTGGCATCCCCGGAGTATGTCAATC
TGCCGATTAATGGTAATGGTAAACAATAATGA

V11A-Y109H

ATGCCACCATATACCGTTGTCTATTTCCCTGCGCGTGGCCGCTGTGCGGCGCTGCGTATGCTGTTGG
CCGACCAGGGTCAGAGCTGGAAAGAAGAAGTTGTTACCGTTGAAACGTGGCAAGAGGGCTCTCTGA
AAGCGAGCTGCCTGTACGGCCAACTGCCGAAGTTTCAGGACGGTGACCTGACGTTGTACCAGAGCA
ACACCATCCTGCGTCACTTGGGTCGCACGTTGGGTCTGTACGGTAAGGACCAGCAAGAGGCCGCAC
TGGTGGACATGGTTAACGATGGTGTCGAGGATCTGCGCTGCAAATACATCAGCCTGATCCATACTAACT
ATGAGGCCGGTAAAGATGATTACGTGAAAGCGCTGCCGGGTCAACTGAAGCCGTTTGAAACCCTGTT
GAGCCAGAACCAGGGTGGCAAGACCTTCATTGTGGGCGATCAAATTTCGTTCGCAGACTATAATCTGC
TGGACCTGCTGCTGATCCACGAAGTGCTGGCTCCGGGCTGTCTGGATGCATTTCCGCTGCTGAGCG
CTTACGTGGGCCGTCTGAGCGCGCGTCCGAAGCTGAAGGCGTTCTTGGCATCCCCGGAGTATGTCA
ATCTGCCGATTAATGGTAATGGTAAACAATAATGA

V11S-Y109H

ATGCCACCATATACCGTTGTCTATTTCCCTAGCCGTGGCCGCTGTGCGGCGCTGCGTATGCTGTTGGC
CGACCAGGGTCAGAGCTGGAAAGAAGAAGTTGTTACCGTTGAAACGTGGCAAGAGGGCTCTCTGAAA
GCGAGCTGCCTGTACGGCCAACTGCCGAAGTTTCAGGACGGTGACCTGACGTTGTACCAGAGCAAC
ACCATCCTGCGTCACTTGGGTCGCACGTTGGGTCTGTACGGTAAGGACCAGCAAGAGGCCGCACTG
GTGGACATGGTTAACGATGGTGTCGAGGATCTGCGCTGCAAATACATCAGCCTGATCCATACTAACTAT
GAGGCCGGTAAAGATGATTACGTGAAAGCGCTGCCGGGTCAACTGAAGCCGTTTGAAACCCTGTTGA
GCCAGAACCAGGGTGGCAAGACCTTCATTGTGGGCGATCAAATTTCGTTCGCAGACTATAATCTGCTG
GACCTGCTGCTGATCCACGAAGTGCTGGCTCCGGGCTGTCTGGATGCATTTCCGCTGCTGAGCGCTT
ACGTGGGCCGTCTGAGCGCGCGTCCGAAGCTGAAGGCGTTCTTGGCATCCCCGGAGTATGTCAATC
TGCCGATTAATGGTAATGGTAAACAATAATGA

V11T-Y109H

ATGCCACCATATACCGTTGTCTATTTCCCTACCCGTGGCCGCTGTGCGGCGCTGCGTATGCTGTTGGC
CGACCAGGGTCAGAGCTGGAAAGAAGAAGTTGTTACCGTTGAAACGTGGCAAGAGGGCTCTCTGAAA
GCGAGCTGCCTGTACGGCCAACTGCCGAAGTTTCAGGACGGTGACCTGACGTTGTACCAGAGCAAC
ACCATCCTGCGTCACTTGGGTCGCACGTTGGGTCTGTACGGTAAGGACCAGCAAGAGGCCGCACTG
GTGGACATGGTTAACGATGGTGTCGAGGATCTGCGCTGCAAATACATCAGCCTGATCCATACTAACTAT
GAGGCCGGTAAAGATGATTACGTGAAAGCGCTGCCGGGTCAACTGAAGCCGTTTGAAACCCTGTTGA
GCCAGAACCAGGGTGGCAAGACCTTCATTGTGGGCGATCAAATTTCGTTCGCAGACTATAATCTGCTG
GACCTGCTGCTGATCCACGAAGTGCTGGCTCCGGGCTGTCTGGATGCATTTCCGCTGCTGAGCGCTT
ACGTGGGCCGTCTGAGCGCGCGTCCGAAGCTGAAGGCGTTCTTGGCATCCCCGGAGTATGTCAATC
TGCCGATTAATGGTAATGGTAAACAATAATGA

V11E-Y109H

ATGCCACCATATACCGTTGTCTATTTCCCTGAGCGTGGCCGCTGTGCGGCGCTGCGTATGCTGTTGGC
CGACCAGGGTCAGAGCTGGAAAGAAGAAGTTGTTACCGTTGAAACGTGGCAAGAGGGCTCTCTGAAA
GCGAGCTGCCTGTACGGCCAACTGCCGAAGTTTCAGGACGGTGACCTGACGTTGTACCAGAGCAAC
ACCATCCTGCGTCACTTGGGTCGCACGTTGGGTCTGTACGGTAAGGACCAGCAAGAGGCCGCACTG
GTGGACATGGTTAACGATGGTGTCGAGGATCTGCGCTGCAAATACATCAGCCTGATCCATACTAACTAT
GAGGCCGGTAAAGATGATTACGTGAAAGCGCTGCCGGGTCAACTGAAGCCGTTTGAAACCCTGTTGA
GCCAGAACCAGGGTGGCAAGACCTTCATTGTGGGCGATCAAATTTCGTTCGCAGACTATAATCTGCTG
GACCTGCTGCTGATCCACGAAGTGCTGGCTCCGGGCTGTCTGGATGCATTTCCGCTGCTGAGCGCTT
ACGTGGGCCGTCTGAGCGCGCGTCCGAAGCTGAAGGCGTTCTTGGCATCCCCGGAGTATGTCAATC
TGCCGATTAATGGTAATGGTAAACAATAATGA

V36R-Y109H

ATGCCACCATATACCGTTGTCTATTTCCCTGTCCGTGGCCGCTGTGCGGCGCTGCGTATGCTGTTGGC
CGACCAGGGTCAGAGCTGGAAAGAAGAAGTTGTTACCCGTGAAACGTGGCAAGAGGGCTCTCTGAA
AGCGAGCTGCCTGTACGGCCAACTGCCGAAGTTTCAGGACGGTGACCTGACGTTGTACCAGAGCAA
CACCATCCTGCGTCACTTGGGTCGCACGTTGGGTCTGTACGGTAAGGACCAGCAAGAGGCCGCACT
GGTGGACATGGTTAACGATGGTGTCGAGGATCTGCGCTGCAAATACATCAGCCTGATCCATACTAACTA

TGAGGCCGGTAAAGATGATTACGTGAAAGCGCTGCCGGGTCAACTGAAGCCGTTTGAAACCCTGTTG
AGCCAGAACCAGGGTGGCAAGACCTTCATTGTGGGCGATCAAATTTCGTTCGCAGACTATAATCTGCT
GGACCTGCTGCTGATCCACGAAGTGCTGGCTCCGGGCTGTCTGGATGCATTTCCGCTGCTGAGCGC
TTACGTGGGCCGTCTGAGCGCGCGTCCGAAGCTGAAGGCGTTCTTGGCATCCCCGGAGTATGTCAAT
CTGCCGATTAATGGTAATGGTAAACAATAA

V36M-Y109H

ATGCCACCATATACCGTTGTCTATTTCCCTGTCCGTGGCCGCTGTGCGGCGCTGCGTATGCTGTTGGC
CGACCAGGGTCAGAGCTGGAAAGAAGAAGTTGTTACCATGGAAACGTGGCAAGAGGGCTCTCTGAA
AGCGAGCTGCCTGTACGGCCAACTGCCGAAGTTTCAGGACGGTGACCTGACGTTGTACCAGAGCAA
CACCATCCTGCGTCACTTGGGTCGCACGTTGGGTCTGTACGGTAAGGACCAGCAAGAGGCCGCACT
GGTGGACATGGTTAACGATGGTGTCGAGGATCTGCGCTGCAAATACATCAGCCTGATCCATACTAACTA
TGAGGCCGGTAAAGATGATTACGTGAAAGCGCTGCCGGGTCAACTGAAGCCGTTTGAAACCCTGTTG
AGCCAGAACCAGGGTGGCAAGACCTTCATTGTGGGCGATCAAATTTCGTTCGCAGACTATAATCTGCT
GGACCTGCTGCTGATCCACGAAGTGCTGGCTCCGGGCTGTCTGGATGCATTTCCGCTGCTGAGCGC
TTACGTGGGCCGTCTGAGCGCGCGTCCGAAGCTGAAGGCGTTCTTGGCATCCCCGGAGTATGTCAAT
CTGCCGATTAATGGTAATGGTAAACAATAA

V36G-Y109H

ATGCCACCATATACCGTTGTCTATTTCCCTGTCCGTGGCCGCTGTGCGGCGCTGCGTATGCTGTTGGC
CGACCAGGGTCAGAGCTGGAAAGAAGAAGTTGTTACCGGTGAAACGTGGCAAGAGGGCTCTCTGAA
AGCGAGCTGCCTGTACGGCCAACTGCCGAAGTTTCAGGACGGTGACCTGACGTTGTACCAGAGCAA
CACCATCCTGCGTCACTTGGGTCGCACGTTGGGTCTGTACGGTAAGGACCAGCAAGAGGCCGCACT
GGTGGACATGGTTAACGATGGTGTCGAGGATCTGCGCTGCAAATACATCAGCCTGATCCATACTAACTA
TGAGGCCGGTAAAGATGATTACGTGAAAGCGCTGCCGGGTCAACTGAAGCCGTTTGAAACCCTGTTG
AGCCAGAACCAGGGTGGCAAGACCTTCATTGTGGGCGATCAAATTTCGTTCGCAGACTATAATCTGCT
GGACCTGCTGCTGATCCACGAAGTGCTGGCTCCGGGCTGTCTGGATGCATTTCCGCTGCTGAGCGC
TTACGTGGGCCGTCTGAGCGCGCGTCCGAAGCTGAAGGCGTTCTTGGCATCCCCGGAGTATGTCAAT
CTGCCGATTAATGGTAATGGTAAACAATAA

V36L-Y109H

ATGCCACCATATACCGTTGTCTATTTCCCTGTCCGTGGCCGCTGTGCGGCGCTGCGTATGCTGTTGGC
CGACCAGGGTCAGAGCTGGAAAGAAGAAGTTGTTACCCTCGAAACGTGGCAAGAGGGCTCTCTGAA
AGCGAGCTGCCTGTACGGCCAACTGCCGAAGTTTCAGGACGGTGACCTGACGTTGTACCAGAGCAA
CACCATCCTGCGTCACTTGGGTCGCACGTTGGGTCTGTACGGTAAGGACCAGCAAGAGGCCGCACT
GGTGGACATGGTTAACGATGGTGTCGAGGATCTGCGCTGCAAATACATCAGCCTGATCCATACTAACTA
TGAGGCCGGTAAAGATGATTACGTGAAAGCGCTGCCGGGTCAACTGAAGCCGTTTGAAACCCTGTTG
AGCCAGAACCAGGGTGGCAAGACCTTCATTGTGGGCGATCAAATTTCGTTCGCAGACTATAATCTGCT
GGACCTGCTGCTGATCCACGAAGTGCTGGCTCCGGGCTGTCTGGATGCATTTCCGCTGCTGAGCGC
TTACGTGGGCCGTCTGAGCGCGCGTCCGAAGCTGAAGGCGTTCTTGGCATCCCCGGAGTATGTCAAT
CTGCCGATTAATGGTAATGGTAAACAATAA

V36K-Y109H

ATGCCACCATATACCGTTGTCTATTTCCCTGTCCGTGGCCGCTGTGCGGCGCTGCGTATGCTGTTGGC
CGACCAGGGTCAGAGCTGGAAAGAAGAAGTTGTTACCAAAGAAACGTGGCAAGAGGGCTCTCTGAA
AGCGAGCTGCCTGTACGGCCAACTGCCGAAGTTTCAGGACGGTGACCTGACGTTGTACCAGAGCAA
CACCATCCTGCGTCACTTGGGTCGCACGTTGGGTCTGTACGGTAAGGACCAGCAAGAGGCCGCACT
GGTGGACATGGTTAACGATGGTGTCGAGGATCTGCGCTGCAAATACATCAGCCTGATCCATACTAACTA
TGAGGCCGGTAAAGATGATTACGTGAAAGCGCTGCCGGGTCAACTGAAGCCGTTTGAAACCCTGTTG
AGCCAGAACCAGGGTGGCAAGACCTTCATTGTGGGCGATCAAATTTCGTTCGCAGACTATAATCTGCT
GGACCTGCTGCTGATCCACGAAGTGCTGGCTCCGGGCTGTCTGGATGCATTTCCGCTGCTGAGCGC
TTACGTGGGCCGTCTGAGCGCGCGTCCGAAGCTGAAGGCGTTCTTGGCATCCCCGGAGTATGTCAAT
CTGCCGATTAATGGTAATGGTAAACAATAA

V36I-Y109H

ATGCCACCATATACCGTTGTCTATTTCCCTGTCCGTGGCCGCTGTGCGGCGCTGCGTATGCTGTTGGC
CGACCAGGGTCAGAGCTGGAAAGAAGAAGTTGTTACCATTGAAACGTGGCAAGAGGGCTCTCTGAAA
GCGAGCTGCCTGTACGGCCAACTGCCGAAGTTTCAGGACGGTGACCTGACGTTGTACCAGAGCAAC
ACCATCCTGCGTCACTTGGGTCGCACGTTGGGTCTGTACGGTAAGGACCAGCAAGAGGCCGCACTG
GTGGACATGGTTAACGATGGTGTCGAGGATCTGCGCTGCAAATACATCAGCCTGATCCATACTAACTAT
GAGGCCGGTAAAGATGATTACGTGAAAGCGCTGCCGGGTCAACTGAAGCCGTTTGAAACCCTGTTGA
GCCAGAACCAGGGTGGCAAGACCTTCATTGTGGGCGATCAAATTTCGTTCGCAGACTATAATCTGCTG
GACCTGCTGCTGATCCACGAAGTGCTGGCTCCGGGCTGTCTGGATGCATTTCCGCTGCTGAGCGCTT
ACGTGGGCCGTCTGAGCGCGCGTCCGAAGCTGAAGGCGTTCTTGGCATCCCCGGAGTATGTCAATC
TGCCGATTAATGGTAATGGTAAACAATAA

V36T-Y109H

ATGCCACCATATACCGTTGTCTATTTCCCTGTCCGTGGCCGCTGTGCGGCGCTGCGTATGCTGTTGGC
CGACCAGGGTCAGAGCTGGAAAGAAGAAGTTGTTACCACCGAAACGTGGCAAGAGGGCTCTCTGAA
AGCGAGCTGCCTGTACGGCCAACTGCCGAAGTTTCAGGACGGTGACCTGACGTTGTACCAGAGCAA
CACCATCCTGCGTCACTTGGGTCGCACGTTGGGTCTGTACGGTAAGGACCAGCAAGAGGCCGCACT
GGTGGACATGGTTAACGATGGTGTCGAGGATCTGCGCTGCAAATACATCAGCCTGATCCATACTAACTA
TGAGGCCGGTAAAGATGATTACGTGAAAGCGCTGCCGGGTCAACTGAAGCCGTTTGAAACCCTGTTG
AGCCAGAACCAGGGTGGCAAGACCTTCATTGTGGGCGATCAAATTTCGTTCGCAGACTATAATCTGCT
GGACCTGCTGCTGATCCACGAAGTGCTGGCTCCGGGCTGTCTGGATGCATTTCCGCTGCTGAGCGC
TTACGTGGGCCGTCTGAGCGCGCGTCCGAAGCTGAAGGCGTTCTTGGCATCCCCGGAGTATGTCAAT
CTGCCGATTAATGGTAATGGTAAACAATAA

V1

ATGCCACCATATACCGTTGTCTATTTCCCTGTCCGTGGCCGCTGTGCGGCGCTGCGTATGCTGTTGGC
CGACCAGGGTCAGAGCTGGAAAGAAGAAGTTGTTAGCGTTGAAACGTGGCTGGAGGGCTCTCTGAAA
AGCAGCTGCCTGTACGGCCAACTGCCGAAGTTTCAGGACGGTGACCTGACGTTGTACCAGAGCAAC
ACCATCCTGCGTCACTTGGGTCGCACGTTGGGTCTGTACGGTAAGGACCAGCGTGAGGCCGCACTG
GTGGACATGGTTAACGATGGTGTCGAGGATCTGCGCTGCAAATACATCAGCCTGATCCATACTAACTAT

GAGGCCGGTAAAGATGATTACGTGAAAGCGCTGCCGGGTCAACTGAAGCCGTTTGAAACCCTGTTGA
GCCAGAACCAGGGTGGCAAGACCTTCATTGTGGGCGATCAAATTTCGTTCGCAGACTATAATCTGCTG
GACCTGCTGCTGATCCACGAAGTGCTGGCTCCGGGCTGTCTGGATGCATTTCCGCTGCTGAGCGCTT
ACGTGGGCCGTCTGAGCGCGCGTCCGAAGCTGAAGGCGTTCTTGGCATCCCCGGAGTATGTCAATC
TGCCGATTAATGGTAATGGTAAACAATAATGA

V2

ATGCCACCATATACCGTTGTCTATTTCCCTGTCCGTGGCCGCTGTGCGGCGCTGCGTATGCTGTTGGC
CGACCAGGGTCAGAGCTGGAAAGAAGAAGTTGTTACCGTTGAAACGTGGATGCAGGGCTCTCTGAAA
AGCAGCTGCCTGTACGGCCAACTGCCGAAGTTTCAGGACGGTGACCTGACGTTGTACCAGAGCAAC
ACCATCCTGCGTCACTTGGGTCGCACGTTGGGTCTGTACGGTAAGGACCAGCAAGAGGCCGCACTG
GTGGACATGGTTAACGATGGTGTCGAGGATCTGCGCTGCAAATACATCAGCCTGATCCATACTAACTAT
GAGGCCGGTAAAGATGATTACGTGAAAGCGCTGCCGGGTCAACTGAAGCCGTTTGAAACCCTGTTGA
GCCAGAACCAGGGTGGCAAGACCTTCATTGTGGGCGATCAAATTTCGTTCGCAGACTATAATCTGCTG
GACCTGCTGCTGATCCACGAAGTGCTGGCTCCGGGCTGTCTGGATGCATTTCCGCTGCTGAGCGCTT
ACGTGGGCCGTCTGAGCGCGCGTCCGAAGCTGAAGGCGTTCTTGGCATCCCCGGAGTATCTGAATC
TGCCGATTAATGGTAATGGTAAACAATAATGA

V3

ATGCCACCATATACCGTTGTCTATTTCCCTGTCCGTGGCCGCTGTGCGGCGCTGCGTATGCTGTTGGC
CGACCAGGGTCAGAGCTGGAAAGAAGAAGTTGTTACCGTTGAAACGTGGCTGGAGGGCCCGCTGAA
AGCGAGCTGCCTGTACGGCCAACTGCCGAAGTTTCAGGACGGTGACCTGACGTTGTACCAGAGCAA
CACCATCCTGCGTCACTTGGGTCGCACGTTGGGTCTGTACGGTAAGGACCAGAAAGAGGCCGCACT
GGTGGACATGGTTAACGATGGTGTCGAGGATCTGCGCTGCAAATACATCAGCCTGATCCATACTAACTA
TGAGGCCGGTAAAGATGATTACGTGAAAGCGCTGCCGGGTCAACTGAAGCCGTTTGAAACCCTGTTG
AGCCAGAACCAGGGTGGCAAGACCTTCATTGTGGGCGATCAAATTTCGTTCGCAGACTATAATCTGCT
GGACCTGCTGCTGATCCACGAAGTGCTGGCTCCGGGCTGTCTGGATGCATTTCCGCTGCTGAGCGC
TTACGTGGGCCGTCTGAGCGCGCGTCCGAAGCTGAAGGCGTTCTTGGCATCCCCGGAGTATCTGAAT
CTGCCGATTAATGGTAATGGTAAACAATAATGA

V4

ATGCCACCATATACCGTTGTCTATTTCCCTGTCCGTGGCCGCTGTGCGGCGCTGCGTATGCTGTTGGC
CGACCAGGGTCAGAGCTGGAAAGAAGAAGTTGTTAGCGTTGAAACGTGGCAACAGGGCTCTCTGAAA
GCGAGCTGCCTGTACGGCCAACTGCCGAAGTTTCAGGACGGTGACCTGACGTTGTACCAGAGCAAC
ACCATCCTGCGTCACTTGGGTCGCACGTTGGGTCTGTACGGTAAGGACCAGAAAGAGGCCGCACTG
GTGGACATGGTTAACGATGGTGTCGAGGATCTGCGCTGCAAATACATCACCCTGATCCATACTAACTAT
GAGGCCGGTAAAGATGATTACGTGAAAGCGCTGCCGGGTCAACTGAAGCCGTTTGAAACCCTGTTGA
GCCAGAACCAGGGTGGCAAGACCTTCATTGTGGGCGATCAAATTTCGTTCGCAGACTATAATCTGCTG
GACCTGCTGCTGATCCACGAAGTGCTGGCTCCGGGCTGTCTGGATGCATTTCCGCTGCTGAGCGCTT
ACGTGGGCCGTCTGAGCGCGCGTCCGAAGCTGAAGGCGTTCTTGGCATCCCCGGAGTATGTCAATC
TGCCGATTAATGGTAATGGTAAACAATAATGA

V5

ATGCCACCATATACCGTTGTCTATTTCCCTGTCCGTGGCCGCTGTGCGGCGCTGCGTATGCTGTTGGC
CGACCAGGGTCAGAGCTGGAAAGAAGAAGTTGTTACCGTTGAAACGTGGATGGAGGGCCCGCTGAA
AGCGAGCTGCCTGTACGGCCAACTGCCGAAGTTTCAGGACGGTGACCTGACGTTGTACCAGAGCAA
CACCATCCTGCGTCACTTGGGTCGCACGTTGGGTCTGTACGGTAAGGACCAGCGTGAGGCCGCACT
GGTGGACATGGTTAACGATGGTGTCGAGGATCTGCGCTGCAAATACATCAGCCTGATCCATACTAACTA
TGAGGCCGGTAAAGATGATTACGTGAAAGCGCTGCCGGGTCAACTGAAGCCGTTTGAAACCCTGTTG
AGCCAGAACCAGGGTGGCAAGACCTTCATTGTGGGCGATCAAATTTCGTTCGCAGACTATAATCTGCT
GGACCTGCTGCTGATCCACGAAGTGCTGGCTCCGGGCTGTCTGGATGCATTTCCGCTGCTGAGCGC
TTACGTGGGCCGTCTGTGCGCGCGTCCGAAGCTGAAGGCGTTCTTGGCATCCCCGGAGTATGTCAAT
CTGCCGATTAATGGTAATGGTAAACAATAATGA

V6

ATGCCACCATATACCGTTGTCTATTTCCCTGTCCGTGGCCGCTGTGCGGCGCTGCGTATGCTGTTGGC
CGACCAGGGTCAGAGCTGGAAAGAAGAAGTTGTTACCGTTGAAACGTGGCAAGAGGGCTCTCTGAAA
GCGAGCTGCCTGTACGGCCAACTGCCGAAGTTTCAGGACGGTGACCTGACGTTGTACCAGAGCAAC
ACCATCCTGCGTCACTTGGGTCGCACGTTGGGTCTGTACGGTAAGGACCAGCGTGAGGCCGCACTG
GTGGACATGGTTAACGATGGTGTCGAGGATCTGCGCAGCAAATACATCACCCTGATCCATACTAACTAT
GAGGCCGGTAAAGATGATTACGTGAAAGCGCTGCCGGGTCAACTGAAGCCGTTTGAAACCCTGTTGA
GCCAGAACCAGGGTGGCAAGACCTTCATTGTGGGCGATCAAATTTCGTTCGCAGACTATAATCTGCTG
GACCTGCTGCTGATCCACGAAGTGCTGGCTCCGGGCTGTCTGGATGCATTTCCGCTGCTGAGCGCTT
ACGTGGGCCGTCTGAGCGCGCGTCCGAAGCTGAAGGCGTTCTTGGCATCCCCGGAGTATCTGAATC
TGCCGATTAATGGTAATGGTAAACAATAATGA

V7

ATGCCACCATATACCGTTGTCTATTTCCCTGTCCGTGGCCGCTGTGCGGCGCTGCGTATGCTGTTGGC
CGACCAGGGTCAGAGCTGGAAAGAAGAAGTTGTTACCGTTGAAACGTGGCAAGAGGGCTCTCTGAAA
AGCAGCTGCCTGTACGGCCAACTGCCGAAGTTTCAGGACGGTGACCTGACGTTGTACCAGAGCAAC
ACCATCCTGCGTCACTTGGGTCGCACGTTGGGTCTGTACGGTAAGGACCAGCAAGAGGCCGCACTG
GTGGACATGGTTAACGATGGTGTCGAGGATCTGCGCTGCAAATACATCACCCTGATCCATACTAACTAT
GAGGCCGGTAAAGATGATTACGTGAAAGCGCTGCCGGGTCAACTGAAGCCGTTTGAAACCCTGTTGA
GCCAGAACCAGGGTGGCAAGACCTTCATTGTGGGCGATCAAATTTCGTTCGCAGACTATAATCTGCTG
GACCTGCTGCTGATCCACGAAGTGCTGGCTCCGGGCTGTCTGGATGCATTTCCGCTGCTGAGCGCTT
ACGTGGGCCGTCTGTGCGCGCGTCCGAAGCTGAAGGCGTTCTTGGCATCCCCGGAGTATGCGAATC
TGCCGATTAATGGTAATGGTAAACAATAATGA

V8

ATGCCACCATATACCGTTGTCTATTTCCCTGTCCGTGGCCGCTGTGCGGCGCTGCGTATGCTGTTGGC
CGACCAGGGTCAGAGCTGGAAAGAAGAAGTTGTTACCGTTGAAACGTGGCTGCAGGGCTCTCTGAAA
GCGAGCTGCCTGTACGGCCAACTGCCGAAGTTTCAGGACGGTGACCTGACGTTGTACCAGAGCAAC
ACCATCCTGCGTCACTTGGGTCGCACGTTGGGTCTGTACGGTAAGGACCCGCAAGAGGCCGCACTG
GTGGACATGGTTAACGATGGTGTCGAGGATCTGCGCTGCAAATACATCAGCCTGATCCATACTAACTAT

GAGGCCGGTAAAGATGATTACGTGAAAGCGCTGCCGGGTCAACTGAAGCCGTTTGAAACCCTGTTGA
GCCAGAACCAGGGTGGCAAGACCTTCATTGTGGGCGATCAAATTTCGTTCGCAGACTATAATCTGCTG
GACCTGCTGCTGATCCACGAAGTGCTGGCTCCGGGCTGTCTGGATGCATTTCCGCTGCTGAGCGCTT
ACGTGGGCCGTCTGAGCGCGCGTCCGAAGCTGAAGGCGTTCTTGGCATCCCCGGAGTATGCGAAT
CTGCCGATTAATGGTAATGGTAAACAATAATGA

V9

ATGCCACCATATACCGTTGTCTATTTCCCTGTCCGTGGCCGCTGTGCGGCGCTGCGTATGCTGTTGGC
CGACCAGGGTCAGAGCTGGAAAGAAGAAGTTGTTAGCGTTGAAACGTGGCAAGAGGGCCCGCTGAA
AGCGAGCTGCCTGTACGGCCAACTGCCGAAGTTTCAGGACGGTGACCTGACGTTGTACCAGAGCAA
CACCATCCTGCGTCACTTGGGTCGCACGTTGGGTCTGTACGGTAAGGACCAGCAAGAGGCCGCACT
GGTGGACATGGTTAACGATGGTGTCGAGGATCTGCGCAGCAAATACATCAGCCTGATCCATACTAACT
ATGAGGCCGGTAAAGATGATTACGTGAAAGCGCTGCCGGGTCAACTGAAGCCGTTTGAAACCCTGTT
GAGCCAGAACCAGGGTGGCAAGACCTTCATTGTGGGCGATCAAATTTCGTTCGCAGACTATAATCTGC
TGGACCTGCTGCTGATCCACGAAGTGCTGGCTCCGGGCTGTCTGGATGCATTTCCGCTGCTGAGCG
CTTACGTGGGCCGTCTGAGCGCGCGTCCGAAGCTGAAGGCGTTCTTGGCATCCCCGGAGTATGCGA
ATCTGCCGATTAATGGTAATGGTAAACAATAATGA

V10

ATGCCACCATATACCGTTGTCTATTTCCCTGTCCGTGGCCGCTGTGCGGCGCTGCGTATGCTGTTGGC
CGACCAGGGTCAGAGCTGGAAAGAAGAAGTTGTTACCGTTGAAACGTGGATGGAGGGCTCTCTGAAA
GCGAGCTGCCTGTACGGCCAACTGCCGAAGTTTCAGGACGGTGACCTGACGTTGTACCAGAGCAAC
ACCATCCTGCGTCACTTGGGTCGCACGTTGGGTCTGTACGGTAAGGACCCGAAAGAGGCCGCACTG
GTGGACATGGTTAACGATGGTGTCGAGGATCTGCGCAGCAAATACATCAGCCTGATCCATACTAACTAT
GAGGCCGGTAAAGATGATTACGTGAAAGCGCTGCCGGGTCAACTGAAGCCGTTTGAAACCCTGTTGA
GCCAGAACCAGGGTGGCAAGACCTTCATTGTGGGCGATCAAATTTCGTTCGCAGACTATAATCTGCTG
GACCTGCTGCTGATCCACGAAGTGCTGGCTCCGGGCTGTCTGGATGCATTTCCGCTGCTGAGCGCTT
ACGTGGGCCGTCTGAGCGCGCGTCCGAAGCTGAAGGCGTTCTTGGCATCCCCGGAGTATGTCAATC
TGCCGATTAATGGTAATGGTAAACAATAATGA

V11

ATGCCACCATATACCGTTGTCTATTTCCCTGTCCGTGGCCGCTGTGCGGCGCTGCGTATGCTGTTGGC
CGACCAGGGTCAGAGCTGGAAAGAAGAAGTTGTTAGCGTTGAAACGTGGCAAGAGGGCTCTCTGAAA
GCGAGCTGCCTGTACGGCCAACTGCCGAAGTTTCAGGACGGTGACCTGACGTTGTACCAGAGCAAC
ACCATCCTGCGTCACTTGGGTCGCACGTTGGGTCTGTACGGTAAGGACCCGCAAGAGGCCGCACTG
GTGGACATGGTTAACGATGGTGTCGAGGATCTGCGCTGCAAATACATCAGCCTGATCCATACTAACTAT
GAGGCCGGTAAAGATGATTACGTGAAAGCGCTGCCGGGTCAACTGAAGCCGTTTGAAACCCTGTTGA
GCCAGAACCAGGGTGGCAAGACCTTCATTGTGGGCGATCAAATTTCGTTCGCAGACTATAATCTGCTG
GACCTGCTGCTGATCCACGAAGTGCTGGCTCCGGGCTGTCTGGATGCATTTCCGCTGCTGAGCGCTT
ACGTGGGCCGTCTGTGCGCGCGTCCGAAGCTGAAGGCGTTCTTGGCATCCCCGGAGTATCTGAATC
TGCCGATTAATGGTAATGGTAAACAATAATGA

V201

ATGCCACCATATACCGTTGTCTATTTCCCTGTCCGTGGCCGCTGTGCGGCGCTGCGTATGCTGTTGGC
CGACCAGGGTCAGAGCTGGAAAGAAGAAGTTGTTAGCGTTGAAACGTGGCTGCAGGGCTCTCTGAAA
GCGAGCTGCCTGTACGGCCAACTGCCGAAGTTTCAGGACGGTGACCTGACGTTGTACCAGAGCAAC
ACCATCCTGCGTCACTTGGGTCGCACGTTGGGTCTGTACGGTAAGGACCCGAAAGAGGCCGCACTG
GTGGACATGGTTAACGATGGTGTCGAGGATCTGCGCTGCAAATACATCACCCTGATCCATACTAACTAT
GAGGCCGGTAAAGATGATTACGTGAAAGCGCTGCCGGGTCAACTGAAGCCGTTTGAAACCCTGTTGA
GCCAGAACCAGGGTGGCAAGACCTTCATTGTGGGCGATCAAATTTCGTTCGCAGACTATAATCTGCTG
GACCTGCTGCTGATCCACGAAGTGCTGGCTCCGGGCTGTCTGGATGCATTTCCGCTGCTGAGCGCTT
ACGTGGGCCGTCTGAGCGCGCGTCCGAAGCTGAAGGCGTTCTTGGCATCCCCGGAGTATGTCAATC
TGCCGATTAATGGTAATGGTAAACAATAATGA

V202

ATGCCACCATATACCGTTGTCTATTTCCCTGTCCGTGGCCGCTGTGCGGCGCTGCGTATGCTGTTGGC
CGACCAGGGTCAGAGCTGGAAAGAAGAAGTTGTTAGCGTTGAAACGTGGCTGCAGGGCTCTCTGAAA
GCGAGCTGCCTGTACGGCCAACTGCCGAAGTTTCAGGACGGTGACCTGACGTTGTACCAGAGCAAC
ACCATCCTGCGTCACTTGGGTCGCACGTTGGGTCTGTACGGTAAGGACCAGAAAGAGGCCGCACTG
GTGGACATGGTTAACGATGGTGTCGAGGATCTGCGCTGCAAATACATCACCCTGATCCATACTAACTAT
GAGGCCGGTAAAGATGATTACGTGAAAGCGCTGCCGGGTCAACTGAAGCCGTTTGAAACCCTGTTGA
GCCAGAACCAGGGTGGCAAGACCTTCATTGTGGGCGATCAAATTTCGTTCGCAGACTATAATCTGCTG
GACCTGCTGCTGATCCACGAAGTGCTGGCTCCGGGCTGTCTGGATGCATTTCCGCTGCTGAGCGCTT
ACGTGGGCCGTCTGTGCGCGCGTCCGAAGCTGAAGGCGTTCTTGGCATCCCCGGAGTATGTCAATC
TGCCGATTAATGGTAATGGTAAACAATAATGA

V203

ATGCCACCATATACCGTTGTCTATTTCCCTGTCCGTGGCCGCTGTGCGGCGCTGCGTATGCTGTTGGC
CGACCAGGGTCAGAGCTGGAAAGAAGAAGTTGTTAGCGTTGAAACGTGGCAACAGGGCTCTCTGAAA
GCGAGCTGCCTGTACGGCCAACTGCCGAAGTTTCAGGACGGTGACCTGACGTTGTACCAGAGCAAC
ACCATCCTGCGTCACTTGGGTCGCACGTTGGGTCTGTACGGTAAGGACCCGAAAGAGGCCGCACTG
GTGGACATGGTTAACGATGGTGTCGAGGATCTGCGCTGCAAATACATCACCCTGATCCATACTAACTAT
GAGGCCGGTAAAGATGATTACGTGAAAGCGCTGCCGGGTCAACTGAAGCCGTTTGAAACCCTGTTGA
GCCAGAACCAGGGTGGCAAGACCTTCATTGTGGGCGATCAAATTTCGTTCGCAGACTATAATCTGCTG
GACCTGCTGCTGATCCACGAAGTGCTGGCTCCGGGCTGTCTGGATGCATTTCCGCTGCTGAGCGCTT
ACGTGGGCCGTCTGTGCGCGCGTCCGAAGCTGAAGGCGTTCTTGGCATCCCCGGAGTATGTCAATC
TGCCGATTAATGGTAATGGTAAACAATAATGA

V204

ATGCCACCATATACCGTTGTCTATTTCCCTGTCCGTGGCCGCTGTGCGGCGCTGCGTATGCTGTTGGC
CGACCAGGGTCAGAGCTGGAAAGAAGAAGTTGTTAGCGTTGAAACGTGGCTGCAGGGCTCTCTGAAA
GCGAGCTGCCTGTACGGCCAACTGCCGAAGTTTCAGGACGGTGACCTGACGTTGTACCAGAGCAAC
ACCATCCTGCGTCACTTGGGTCGCACGTTGGGTCTGTACGGTAAGGACCCGAAAGAGGCCGCACTG
GTGGACATGGTTAACGATGGTGTCGAGGATCTGCGCTGCAAATACATCACCCTGATCCATACTAACTAT

GAGGCCGGTAAAGATGATTACGTGAAAGCGCTGCCGGGTCAACTGAAGCCGTTTGAAACCCTGTTGA
GCCAGAACCAGGGTGGCAAGACCTTCATTGTGGGCGATCAAATTTCGTTCGCAGACTATAATCTGCTG
GACCTGCTGCTGATCCACGAAGTGCTGGCTCCGGGCTGTCTGGATGCATTTCCGCTGCTGAGCGCTT
ACGTGGGCCGTCTGTGCGCGCGTCCGAAGCTGAAGGCGTTCTTGGCATCCCCGGAGTATGTCAATC
TGCCGATTAATGGTAATGGTAAACAATAATGA

V205

ATGCCACCATATACCGTTGTCTATTTCCCTGTCCGTGGCCGCTGTGCGGCGCTGCGTATGCTGTTGGC
CGACCAGGGTCAGAGCTGGAAAGAAGAAGTTGTTACCGTTGAAACGTGGCAACAGGGCTCTCTGAAA
GCGAGCTGCCTGTACGGCCAACTGCCGAAGTTTCAGGACGGTGACCTGACGTTGTACCAGAGCAAC
ACCATCCTGCGTCACTTGGGTCGCACGTTGGGTCTGTACGGTAAGGACCCGAAAGAGGCCGCACTG
GTGGACATGGTTAACGATGGTGTCGAGGATCTGCGCTGCAAATACATCACCCTGATCCATACTAACTAT
GAGGCCGGTAAAGATGATTACGTGAAAGCGCTGCCGGGTCAACTGAAGCCGTTTGAAACCCTGTTGA
GCCAGAACCAGGGTGGCAAGACCTTCATTGTGGGCGATCAAATTTCGTTCGCAGACTATAATCTGCTG
GACCTGCTGCTGATCCACGAAGTGCTGGCTCCGGGCTGTCTGGATGCATTTCCGCTGCTGAGCGCTT
ACGTGGGCCGTCTGTGCGCGCGTCCGAAGCTGAAGGCGTTCTTGGCATCCCCGGAGTATGTCAATC
TGCCGATTAATGGTAATGGTAAACAATAATGA

V206

ATGCCACCATATACCGTTGTCTATTTCCCTGTCCGTGGCCGCTGTGCGGCGCTGCGTATGCTGTTGGC
CGACCAGGGTCAGAGCTGGAAAGAAGAAGTTGTTACCGTTGAAACGTGGCTGCAGGGCTCTCTGAAA
GCGAGCTGCCTGTACGGCCAACTGCCGAAGTTTCAGGACGGTGACCTGACGTTGTACCAGAGCAAC
ACCATCCTGCGTCACTTGGGTCGCACGTTGGGTCTGTACGGTAAGGACCCGAAAGAGGCCGCACTG
GTGGACATGGTTAACGATGGTGTCGAGGATCTGCGCTGCAAATACATCACCCTGATCCATACTAACTAT
GAGGCCGGTAAAGATGATTACGTGAAAGCGCTGCCGGGTCAACTGAAGCCGTTTGAAACCCTGTTGA
GCCAGAACCAGGGTGGCAAGACCTTCATTGTGGGCGATCAAATTTCGTTCGCAGACTATAATCTGCTG
GACCTGCTGCTGATCCACGAAGTGCTGGCTCCGGGCTGTCTGGATGCATTTCCGCTGCTGAGCGCTT
ACGTGGGCCGTCTGTGCGCGCGTCCGAAGCTGAAGGCGTTCTTGGCATCCCCGGAGTATGTCAATC
TGCCGATTAATGGTAATGGTAAACAATAATGA

V401

ATGCCACCATATACCGTTGTCTATTTCCCTGTCCGTGGCCGCTGTGCGGCGCTGCGTATGCTGTTGGC
CGACCAGGGTCAGAGCTGGAAAGAAGAAGTTGTTACCGTTGAAACGTGGCAAGAGGGCTCTCTGAAA
GCGAGCTGCCTGTACGGCCAACTGCCGAAGTTTCAGGACGGTGACCTGACGTTGTACCAGAGCAAC
ACCATCCTGCGTCACTTGGGTCGCACGTTGGGTCTGTACGGTAAGGACCAGCGTGAGGCCGCACTG
GTGGACATGGTTAACGATGGTGTCGAGGATCTGCGCTGCAAATACATCAGCCTGATCCATACTAACTAT
GAGGCCGGTAAAGATGATTACGTGAAAGCGCTGCCGGGTCAACTGAAGCCGTTTGAAACCCTGTTGA
GCCAGAACCAGGGTGGCAAGACCTTCATTGTGGGCGATCAAATTTCGTTCGCAGACTATAATCTGCTG
GACCTGCTGCTGATCCACGAAGTGCTGGCTCCGGGCTGTCTGGATGCATTTCCGCTGCTGAGCGCTT
ACGTGGGCCGTCTGAGCGCGCGTCCGAAGCTGAAGGCGTTCTTGGCATCCCCGGAGTATGTCAATC
TGCCGATTAATGGTAATGGTAAACAATAATGA

Rat GST P1-1

ATGCCCCCCTACACCATCGTGTACTTCCCCGTGAGGGGCAGGTGCGAGGCCACCAGGATGCTGC
TGGCCGACCAGGGCCAGAGCTGGAAGGAGGAGGTGGTGACCATCGACGTGTGGCTGCAGGGCA
GCCTGAAGAGCACCTGCCTGTACGGCCAGCTGCCCAAGTTCGAGGACGGCGACCTGACCCTGTA
CCAGAGCAACGCCATCCTGAGGCACCTGGGCAGGAGCCTGGGCCTGTACGGCAAGGACCAGAA
GGAGGCCGCCCTGGTGGACATGGTGAACGACGGCGTGGAGGACCTGAGGTGCAAGTACGGCAC
CCTGATCTACACCAACTACGAGAACGGCAAGGACGACTACGTGAAGGCCCTGCCCGGCCACCTG
AAGCCCTTCGAGACCCTGCTGAGCCAGAACCAGGGCGGCAAGGCCTTCATCGTGGGCAACCAG
ATCAGCTTCGCCGACTACAACCTGCTGGACCTGCTGCTGGTGCACCAGGTGCTGGCCCCCGGCT
GCCTGGACAACTTCCCCCTGCTGAGCGCCTACGTGGCCAGGCTGAGCGCCAGGCCCAAGATCA
AGGCCTTCCTGAGCAGCCCCGACCACCTGAACAGGCCCATCAACGGCAACGGCAAGCAG

Dog GST P1-1

ATGCCTCCGTACACCATTACCTATTTCCCGGTCCGTGGTCGCTGCGAAGCGATGCGCATGCTGCTGG
CGGATCAGGGTCAGAGCTGGAAAGAGGAAGTCGTCACGATGGAAACCTGGATGAAAGGCTCCCTGA
AGGCCAGCTGTCTGTATGGTCAGCTGCCGAAGTTCCAAGATGGCGATCTGACCCTGTACCAATCTAAC
GCAATTCTGCGTCACCTGGGCCGCTCCCTGGGCCTCTATGGTAAAGACCAGCAAGAGGCAGCGTTG
TTAGACGTTGTTAATGACGGCGTTGAAGATCTGCGTTGTAAATACGCACTGCTGATTTACACGAACTATG
AGGCCGGCAAAGAGGAATATGTTAAGGCCCTGCCGGGTTACCTGAAACCGTTCGAGACTCTGTTGAG
CCAGAACGAGGGTGGCCAAGCGTTTATCGTGGGTAACCAGATTAGCTTTGCGGATTACAATCTGCTTG
ACTTGCTGTTAATCCACCAAGTCCTGGCTCCGAGCTGCCTGGACTCGTTCCCGCTGTTGAGCGCCTA
CGTGGCGCGTCTGAGCGCACGTCCGAAGCTGAAGGCTTTTCTGAGCAGCCCGGAACATGTGAACC
GTCCAATCAATGGTAATGGTAAGCAATAATAA

Mouse GST P1-1

ATGCCTCCGTACACCATTGTGTATTTCCCGGTCCGTGGTCGCTGCGAAGCGATGCGCATGCTGCTGG
CGGATCAGGGTCAGAGCTGGAAAGAGGAAGTCGTCACGATCGATACCTGGATGCAAGGCTTGCTGAA
GCCGACCTGTCTGTATGGTCAGCTGCCGAAGTTCGAAGATGGCGATCTGACCCTGTACCAATCTAACG
CAATTCTGCGTCACCTGGGCCGCTCCCTGGGCCTCTATGGTAAAAACCAGCGCGAGGCAGCGCAA
ATGGACATGGTTAATGACGGCGTTGAAGATCTGCGTGGTAAATACGTTACGCTGATTTACACGAACTATG
AGAACGGCAAAAATGACTATGTTAAGGCCCTGCCGGGTCATCTGAAACCGTTCGAGACTCTGTTGAGC
CAGAACCAGGGTGGCAAAGCGTTTATCGTGGGTGACCAGATTAGCTTTGCGGATTACAATCTGCTTGA
CTTGCTGTTAATCCACCAAGTCCTGGCTCCGGGTTGCCTGGACAATTTCCCGCTGTTGAGCGCCTAC
GTGGCGCGTCTGAGCGCACGTCCGAAGATCAAGGCTTTTCTGAGCAGCCCGGAACATGTGAACCGT
CCAATCAATGGTAATGGTAAGCAATAATAA

Mouse GST P2-2

ATGCCTCCGTACACCATTGTGTATTTCCCGAGCCCGGGTCGCTGCGAAGCGATGCGCATGCTGCTG
GCGGATCAGGGTCAGAGCTGGAAAGAGGAAGTCGTCACGATCGATACCTGGATGCAAGGCTTGCTG
AAGCCGACCTGTCTGTATGGTCAGCTGCCGAAGTTCGAAGATGGCGATCTGACCCTGTACCAATCTAA
CGCAATTCTGCGTCACCTGGGCCGCTCCCTGGGCCTCTATGGTAAAAACCAGCGCGAGGCAGCGC
AAGTTGACATGGTTAATGACGGCGTTGAAGATCTGCGTGGTAAATACGGTACGATGATTTACCGTAACTA
TGAGAACGGCAAAAATGACTATGTTAAGGCCCTGCCGGGTCATCTGAAACCGTTCGAGACTCTGTTGA
GCCAGAACCAGGGTGGCAAAGCGTTTATCGTGGGTGACCAGATTAGCTTTGCGGATTACAATCTGCTT

GACTTGCTGTTAATCCACCAAGTCCTGGCTCCGGGTTGCCTGGACAATTTCCCGCTGTTGAGCGCCTA
CGTGGCGCGTCTGAGCGCACGTCCGAAGATCAAGGCTTTTCTGAGCAGCCCGGAACATGTGAACCG
TCCAATCAATGGTAATGGTAAGCAATAATAA
